# Immigrant and Racialized Populations’ Cumulative Exposure to Discrimination and Associations with Long-Term Conditions During COVID-19: A Nationwide Large-Scale Study in Canada

**DOI:** 10.1007/s40615-024-02074-1

**Published:** 2024-07-17

**Authors:** Shen (Lamson) Lin

**Affiliations:** 1 Department of Social and Behavioural Sciences, City University of Hong Kong, Hong Kong SAR, China; 2 Factor-Inwentash Faculty of Social Work, University of Toronto, Toronto, Canada; 3 Oxford Institute of Population Ageing, University of Oxford, Oxford, United Kingdom

**Keywords:** Intersectionality, Immigrant health, Health inequality, Racism, Chronic stress

## Abstract

**Background:**

This cross-sectional study examines associations between the race-migration nexus, cumulative exposure to intersectional discrimination (2 years before and during the COVID-19 pandemic), and long-term conditions.

**Methods:**

A nationwide self-selected sample (*n* = 32,605) was obtained from a Statistics Canada’s Crowdsourcing online survey from August 4 to 24, 2020. Binary and multinomial logistic regression models were used to examine disparities by the race-migration nexus in accumulative experiences of multiple situations- and identity-based discrimination and their relations with long-term conditions, after controlling for sociodemographic covariates.

**Results:**

During the pandemic, discrimination stemming from racialization — such as race/skin color (24.4% vs 20.1%) and ethnicity/culture (18.5% vs 16.5%) — and cyberspace (34.1% vs 29.8%) exaggerated relative to pre-pandemic period; compared to Canadian-born (CB) whites, the likelihood of experiencing multiple discrimination increased alongside the domains of discrimination being additively intersected (e.g., identity-based, all *p*’s < 0.001) among CB racialized minorities (ORs 2.08 to 11.78), foreign-born (FB) racialized minorities (ORs 1.99 to 12.72), and Indigenous populations (ORs 1.62 to 8.17), except for FB whites (*p* > 0.01); dose-response relationships were found between cumulative exposure to multiple discrimination and odds of reporting long-term conditions (*p*’s < 0.001), including seeing (ORs 1.63 to 2.99), hearing (ORs 1.83 to 4.45), physical (ORs 1.66 to 3.87), cognitive (ORs 1.81 to 3.79), and mental health–related impairments (ORs 1.82 to 3.41).

**Conclusions:**

Despite a universal health system, Canadians who are CB/FB racialized and Indigenous populations, have a higher prevalence of cumulative exposure to different aspects of discrimination that are associated with multiple long-term conditions during the COVID-19 pandemic. Equity-driven solutions are needed to tackle upstream determinants of health inequalities through uprooting intersectional discrimination faced by racialized and immigrant communities.

**Supplementary Information:**

The online version contains supplementary material available at 10.1007/s40615-024-02074-1.

## Introduction

Growing evidence has demonstrated the overrepresentation of racialized, low-income, and other socially marginalized populations among cases of and deaths from COVID-19 across the globe [1–3]. What the COVID-19 pandemic brings to the forefront is that both exposure to health hazards and access to health-enhancing resources are fundamentally different based on race, migration, and many other social determinants of health (SDoH) [4, 5]. Meanwhile, the pandemic not only has sparked a big rise in COVID-19-associated discrimination that disproportionately affected racialized and immigrant minorities but also exacerbated pre-existing resentment, hate, and violence against these marginalized communities [6–8]. According to recent pandemic-specific population-based studies, immigrants were twice as likely as Canadian-born peers to experience COVID-19-related stigma [9] and such excess fear of being targeted as risky others was attributable to the framing of “foreign virus” that mistakenly blaming the pandemic on foreign-born “others” [10]. Immigrants who experienced pandemic-triggered job insecurity such as business closure and layoff were the most-at-risk group of developing severe loneliness (e.g., feeling isolated from others) during the COVID-19 lockdown period in Canada [11].

Population health science suggests that discrimination — the unfair or prejudicial treatment of individuals based on social markers — is a crucial mechanism that (re)produce systematic inequalities in the daily circumstances and lived experiences that shape the distribution of health and disease [12–14]. The distal impact of chronic stress resulting from social vulnerabilities (also known as adverse SDoH) and discrimination could be transmitted “under the skin” through many downstream channels [15–17], including dysregulation of physiological systems, immune and inflammation response, and underlying chronic conditions, that were associated with critical COVID-19 illness [18]. While health inequalities are well documented in relation to race/ethnicity and migration, prior research has typically examined these health differences separately and treated immigrants with diverse racial backgrounds as a monolithic population [19]. The intersection between racialization and immigration (e.g., the race-migration nexus) has been largely overlooked in epidemiology [20]. As such, the intersectionality theory has much to offer as it unpacks various minority struggles that are often obscured within a liberal discourse of egalitarianism. Intersectionality is a theoretical paradigm that originated from Black feminist theory [21], which later extends to other health and social disciplines with the notion that individuals’ numerous social positions (e.g., race, age, gender, sexuality, nationality, income, education) simultaneously affect the human experience, resulting in unique combinations of discrimination, marginalization, and privilege. Following this line of inquiry, there have been increasing calls for population health research to adopt an intersectionality framework in understanding intersectional experiences of discrimination through various approaches, including attributions to different grounds [22], multiple minority stress [23], various dimensions of stereotype [24], and discrimination index [25].

**Fig. 1:**
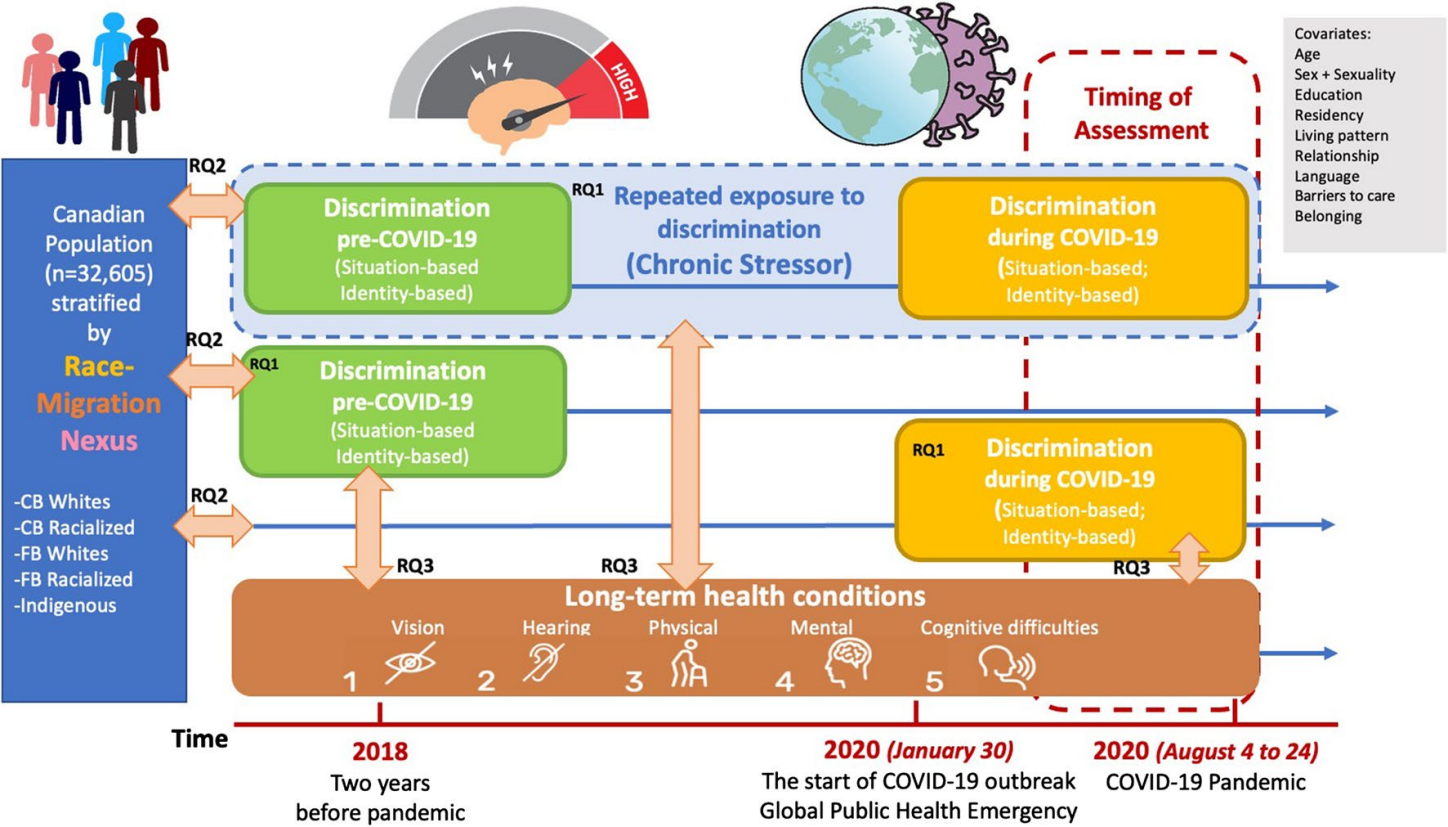
Conceptual framework of understanding relations between race-migration nexus, exposure to discrimination, and long-term conditions (key measures) in Canada. Notes: CB, Canadian-born; FB, foreign-born (immigrants)

Immigrants make up one-fifth (22%) of the total Canadian population and their countries of origin have primarily shifted from the Global North to the Global South, representing diverse ethnicities, culture, languages, and religions from non-European countries (e.g., China, India) [9, 26, 27]. However, in migrant health literature, racialized immigrant communities were largely neglected due to the ethnocentric assimilation paradigm [27], and when given attention, they were often compared with native-born counterparts of the same ethnic origin in the hosting countries [26], instead of using the ethnic majority as the reference group [27]. This intra-ethnic group comparison would give rise to methodological bias, because racialized immigrants may bear greater susceptibility to unfair treatments in inter-ethnic encounters such as limited social mobility, nativism, and xenophobia [28, 29]. In addition, the Canada’s multiculturalism ideology further overshadows the manifestation of structural racism and racial profiling in the postcolonial society [29, 30], rendering the invisibility of “visible minorities” in Canadian health research [31]. Less is known about how the intersectional experience of multiple discrimination unfolds among diverse communities across the race-migration nexus during the COVID-19 pandemic in Canada. Furthermore, there is limited data regarding the linkage between perceived discrimination and chronic conditions in the COVID-19 times [32]. Thus, informed by prior studies on race-migration nexus [26, 27, 33, 34], a new conceptual framework was developed, as shown in Fig. 1, to unpack nuanced racial and immigration-based disparities in discrimination-health relationships, with a particular focus on population-level dynamics between race/ism, repeated exposure to discrimination, and self-reported long-term conditions. This framework is tailored to the data collection process to reflect the retrospective time frame of key variables measured by the survey questionnaire. The current study preferred the term “racialized” by society over other terminologies (e.g., ethnocultural/visible minority, people of color) to acknowledge race as a social construct resulting from racialization processes [26, 27]. Reducing inequalities of outcome by eliminating discriminatory practices is central to the attainment of the United Nation’s 2030 Agenda for Sustainable Development Goals (SDGs), especially SGD 3 and SGD 10. This study serves as a nationwide assessment in Canada to investigate three major equity-driven research questions (RQ) below:

1. What are the prevalence and changing patterns of cumulative exposure to discrimination based on multiple marginalized identities/situations across diverse racialized/immigrant groups before and during the COVID-19 pandemic?
2. Are racialized and immigrant minorities more likely to experience cumulative exposure to multiple discrimination (during the COVID-19 pandemic) as well as repeated exposure to discrimination at two points in time (before and during COVID-19), compared to the Canadian-born white population?
3. Are multiple forms of perceived discrimination (during the COVID-19 pandemic) and repeated exposure to discrimination (as chronic stressor) both associated with various types of long-term health conditions, after adjusting for sociodemographic characteristics?

## Methods

### Data Source and Study Context

This study used data from Statistics Canada’s Crowdsourcing Data: Impacts of COVID-19 on Canadians—Experiences of Discrimination (ICCED) [35]. Based on a self-selection approach, respondents were recruited using an open advertisement on the Internet and survey data were collected via online questionnaires between August 4 and 24, 2020 (around seven months after the COVID-19 outbreak as a public health emergency declared by the World Health Organization on January 30, 2020). Participation in this crowdsourcing initiative was voluntary. The target population comprises all Canadians aged 15 and up who live in one of the ten provinces or three territories in Canada. Although it did not adopt a probabilistic approach to collecting data, crowdsourcing data could provide a timely snapshot capturing the population’s health during the COVID-19 pandemic [36]. Unlike other surveys conducted by Statistics Canada, a survey weight cannot be calculated through crowdsourcing. Rather, benchmarking techniques were applied to correct for unbalanced responses across sociodemographic characteristics based on June 2020 demographic projections. Similar to the post-stratification approach, basic adjustments to known control totals (i.e., number of people by province, age group, sex, and visible minority status) were adopted to mitigate bias [35]. To ensure the rigor and data quality, Statistics Canada used multiple strategies, including basic data verifications (e.g., valid postal code), outlier detection, out-of-scope records, collection problems (e.g., participants answered the survey more than one), and comparisons between ICCED with other reliable probabilistic sources (e.g., Canadian Perspective Survey Series). A detailed methodology for this crowdsourcing survey can be found in Statistics Canada’s report [35, 36]. Examples of the survey questions for key variables in this study could be found in the Supplemental material online (Supplement \* MERGEFORMAT 1.

### Ethical Considerations

The analyses were based on the public-use microdata files (PUMF) available to both Canadian and international researchers via Statistics Canada’s Data Liberation Initiative. The public-use data are completely de-identified with necessary suppression methods to protect confidentiality; thus, according to the Tri-Council Policy Statement: Ethical Conduct for Research Involving Humans — TCPS 2 (2018), this study is considered non-human subject research that does not require institutional ethics review.

### Race-Migration Nexus

Consistent with prior studies [26, 27], the race-migration nexus was conceptualized as a key structural driver of inequalities (i.e., a proxy of structural forces) in this study to capture the dual stratification process resulting from racialization and migration experiences in shaping power differentials and resource allocation [20, 30]. Based on the questions about “ethnic origin” (i.e., ethnic or cultural ancestry) and “country of origin,” the race-migration nexus was coded into five social positionings: Canadian-born (CB) white (reference), CB racialized minorities (CB non-white), foreign-born (FB) white, racialized immigrants (FB non-white), and Indigenous populations (i.e., First Nations, Métis or Inuk). For simplicity in presenting the figures and tables, the terms non-whites and racialized groups were used interchangeably. This variable of race-migration nexus was regarded as more than individual attributes but as a product of power structures as well as system of stratification that rank people into social hierarchies, thus (re)distribuinge social determinants of health [20]. CB whites were treated as the reference group as it reflects a social location of privilege that shapes human experiences in a white settler society [26], where whiteness, legacies of colonialism, and the related policing of ethnic hierarchy may continue to perpetuate white superiority [29].

### Perceived Discrimination

In this survey, discrimination refers to treating a person or a group unfairly because of who they are, or because they possess certain characteristics. Respondents were asked two questions about subjective assessments of discrimination: (1) whether they had experienced discrimination or been treated unfairly by others in Canada because of their social identities (attributed reasons) and (2) in what types of situations they experienced discrimination. These two questions were asked repeatedly in the context of “two years before the COVID-19 pandemic” (e.g., the year of 2018) and the context “during the COVID-19 pandemic” (e.g., the year of 2020). For the identity-based discrimination, eleven options of social identities were listed, including 1, indigeneity; 2, ethnicity/culture; 3, race/skin color; 4, religion; 5, language; 6, accent; 7, physical appearance; 8, sex/sexual orientation/gender expression; 9, age; 10, physical/mental disability; and 11, other unspecified reason. For situation-based discrimination, twelve options were listed to reflect “institutionally mediated experiences of discrimination” wherein resources may be unequally distributed between social groups, including 1, store/bank/restaurant; 2, at school; 3, at work; 4, housing; 5, police; 6, court; 7, social gathering; 8, crossing the Canadian borders; 9, on the Internet; 10, on public transport; 11, in a public area; and 12, other unspecified reason. Respondents were asked to choose all that apply. In the current study, the measures of identity-based and situation-based discrimination were dichotomized (Yes/No), indicating any exposure to discrimination. In addition, as with prior studies [29], in order to measure cumulative experiences of discrimination, we then created addictive variables that combine different domains of multiple discrimination. and they were categorized into five levels: 0, no exposure; 1, exposure to one domain (identity/situation); 2, exposure to two domains; 3, exposure to three domains; 4, exposure to four or more domains. Lastly, another variable, namely repeated exposure to discrimination (representing chronic stressor across two periods), was coded into four levels: 0, no exposure; 1, exposure only before the pandemic; 2, exposure only during the pandemic; 3, repeated exposure before and during the pandemic. This indicator (the second category) could isolate respondents with new experiences of discrimination during the pandemic and see if they face (or not) excess disparities in new cases of victimization triggered by the pandemic.

### Long-Term Conditions

Respondents were asked if they have any of the following difficulties or long-term conditions that have lasted or are expected to last for six or more months: (1) vision impairment (difficulty seeing, even when wearing glasses or contact lenses), (2) hearing impairment (difficulty hearing, even when using a hearing aid or cochlear implant), (3) physical impairment (difficulty walking, using stairs, using your hands or fingers or doing other physical activities), (4) cognitive problems (difficulty learning, remembering or concentrating), (5) mental health problems (emotional, psychological or mental health conditions such as anxiety, depression, bipolar disorder, substance abuse, anorexia), and (6) other health problems. Focusing on the component of activity limitations (instead of specific chronic diseases and conditions), four out of these six options were derived from the The Washington Group Short Set on Functioning (WG-SS) instruments, which reflects the World Health Organization (WHO)’s International Classification of Functioning, Disability, and Health (ICF) to allow international comparison. These six conditions also represented various domains of "intrinsic capacity" (a crucial marker for healthy longevity monitoring) as defined by WHO's positive model of healthy ageing, including individuals' "ability to walk, think, see, hear and remember". Respondents were asked to choose all options that apply (Yes vs No). These six conditions were coded seperately into six binary variables.

### Covariates

Covariates included age, sex, education, urban–rural residency, living arrangement, sexuality, marital status, primary language at home, barriers to health care access during the pandemic, and community belonging (weak versus strong). These covariates were selected into the statistical model based on a knowledge synthesis paper, a narrative review which delineated a socio-ecological conceptual framework of understanding health care inequity shaped by the race-migration nexus in Canada [4]. Detailed response options for these covariate variables can be found in sample characteristics (Table 1). Barriers to health care were measured by a question asking respondents if they personally experienced problems accessing the following services since the beginning of the COVID-19 pandemic: surgery, diagnostic test, appointment with a family doctor, specialist services, rehabilitative care, dental care, mental health care, medical treatment, natural medicine, and urgent care. Response categories were grouped by the ICCED into four levels: (1) no accessibility issues in any service, (2) accessibility issues with one service, (3) accessibility issues with multiple services, and (4) no services needed.

**Table 1:** Sample characteristics stratified by race-migration nexus in Canada, ICCED, August 2020 (*N* = 32,605)

	Average	CB whites	CB non-whites	FB whites	FB non-whites	Indigenous	*X*^2^
	*n* = 32,605	*n* = 24,412	*n* = 1865	*n* = 2300	*n* = 2887	*n* = 1141	Sig
	100%	74.9%	5.7%	7.1.%	8.9%	3.5%	
Age							< 0.001
15–44	49.7%	48.1%	75.1%	34.8%	57.4%	52.8%	
45 +	50.3%	51.9%	24.9%	65.2%	42.6%	47.2%	
Sex							< 0.001
Male	28.4%	27.1%	29.1%	32.4%	36.5%	25.2%	
Female	71.6%	72.9%	70.9%	67.6%	63.5%	74.8%	
Education							< 0.001
< University	34.3%	37.8%	22.0%	24.7%	13.7%	50.0%	
Attended university	65.7%	62.2%	78.0%	75.3%	86.3%	50.0%	
Municipality/Residence							< 0.001
Rural	9.3%	10.8%	1.3%	8.2%	0.8%	14.0%	
Urban	89.0%	87.4%	97.5%	90.7%	97.5%	82.2%	
Not stated	1.7%	1.8%	1.2%	1.1%	1.6%	3.8%	
Living arrangement							< 0.001
Living alone	15.8%	16.2%	14.1%	16.4%	13.3%	16.4%	
Multiple persons; no children	45.1%	45.6%	43.4%	49.7%	39.3%	42.8%	
Multiple persons; have children	39.1%	38.2%	42.5%	33.9%	47.4%	40.8%	
Sexual orientation							< 0.001
LGBTQ2	11.8%	11.8%	13.3%	10.8%	8.2%	19.3%	
Non-LGBTQ2	88.2%	88.2%	86.7%	89.2%	91.8%	80.7%	
Marital status							< 0.001
Married/common law	68.4%	69.5%	56.1%	73.1%	66.5%	60.7%	
Not married	31.6%	30.5%	43.9%	26.9%	33.5%	39.3%	
Language spoken at home							< 0.001
English and French	2.4%	2.2%	2.4%	2.4%	4.0%	3.6%	
English	79.9%	81.2%	86.8%	80.1%	61.7%	86.4%	
French	14.3%	16.4%	5.5%	8.3%	8.8%	9.6%	
Other language(s)	3.4%	0.2%	5.4%	9.2%	25.5%	0.4%	
Health care access							< 0.001
No access barrier	33.6%	33.2%	36.6%	35.3%	36.9%	25.2%	
Barrier with 1 service	19.2%	19.7%	16.6%	19.1%	18.5%	16.0%	
Barrier with ≥ 2 services	36.2%	36.5%	32.4%	35.9%	31.3%	49.2%	
No services needed	11.0%	10.7%	14.4%	9.7%	13.3%	9.6%	
Trust in health care system							< 0.001
Have trust	86.9%	88.0%	85.0%	88.0%	83.6%	72.0%	
No trust	13.1%	12.0%	15.0%	12.0%	16.4%	28.0%	
Community belonging							< 0.001
Weak belonging	29.5%	28.1%	35.5%	29.0%	33.9%	39.4%	
Strong belonging	69.4%	70.8%	63.7%	69.7%	65.1%	59.6%	
Not state	1.1%	1.1%	0.8%	1.3%	1.0%	1.0%	
Canada belonging							< 0.001
Weak belonging	11.9%	10.4%	15.8%	12.0%	16.3%	26.7%	
Strong belonging	86.9%	88.5%	83.4%	86.8%	82.1%	72.7%	
Not state	1.1%	1.1%	0.8%	1.2%	1.6%	0.5%	
Disability status							< 0.001
LTC; with disability	13.9%	14.8%	10.6%	10.8%	6.6%	26.0%	
LTC; no disability	35.2%	35.5%	36.9%	34.1%	31.7%	36.6%	
No LTC; with disability	0.9%	0.9%	0.9%	0.8%	0.9%	1.1%	
No LTC; no disability	49.9%	48.8%	51.6%	54.3%	60.8%	36.2%	
Long-term conditions (LTC)							< 0.001
No health condition	50.8%	49.7%	52.5%	55.1%	61.7%	37.3%	
Have health condition(s)	49.2%	50.3%	47.5%	44.9%	38.3%	62.7%	
1 condition	28.6%	29.3%	27.3%	28.5%	24.0%	28.4%	< 0.001
2 conditions	12.8%	13.1%	12.7%	10.7%	9.7%	18.4%	
3 conditions	5.1%	5.2%	5.3%	4.1%	3.3%	9.4%	
4 conditions	2.7%	2.8%	2.3%	1.7%	1.4%	6.5%	
Specific LTC condition							
Seeing difficulties	8.3%	8.2%	9.4%	7.1%	7.8%	12.0%	< 0.001
Hearing difficulties	2.4%	2.6%	1.6%	2.9%	0.8%	2.4%	< 0.001
Physical act difficulties	10.2%	10.7%	7.2%	9.9%	5.2%	18.0%	< 0.001
Cognitive difficulties	9.4%	9.4%	10.1%	6.3%	7.9%	16.0%	< 0.001
Mental health conditions	28.5%	29.1%	31.3%	21.7%	21.6%	41.4%	< 0.001
Other conditions	22.1%	22.8%	18.2%	21.5%	15.4%	31.6%	< 0.001
Situation-based discrimination							
Exposure during COVID-19	27.0%	22.4%	51.5%	21.7%	46.1%	47.5%	< 0.001
1 situation	12.9%	11.7%	18.8%	12.0%	18.9%	14.9%	< 0.001
2 situations	6.9%	5.6%	13.6%	5.3%	11.6%	13.1%	
3 situations	3.5%	2.6%	8.3%	2.2%	7.2%	7.8%	
≥ 4 situations	3.7%	2.4%	10.7%	2.1%	8.4%	11.7%	
Exposure before COVID-19	44.1%	39.2%	69.4%	38.1%	64.7%	67.4%	< 0.001
1 situation	16.4%	15.8%	16.1%	17.3%	20.6%	16.8%	< 0.001
2 situations	10.5%	9.2%	15.5%	10.9%	15.9%	14.7%	
3 situations	7.1%	6.4%	12.7%	4.8%	10.3%	10.7%	
≥ 4 situations	10.1%	7.8%	25.0%	5.2%	17.8%	25.2%	
Identity-based discrimination							
Exposure during COVID-19	27.0%	22.4%	51.5%	21.7%	46.1%	47.5%	< 0.001
1 identity	13.9%	13.5%	17.2%	12.0%	15.9%	16.8%	< 0.001
2 identities	6.9%	5.3%	16.0%	5.6%	13.1%	11.4%	
3 identities	3.4%	2.2%	9.0%	2.6%	8.2%	9.6%	
≥ 4 identities	2.8%	1.3%	9.3%	1.6%	8.9%	9.6%	
Exposure before COVID-19	44.1%	39.2%	69.4%	38.1%	64.7%	67.4%	< 0.001
1 identity	19.4%	20.1%	15.1%	18.1%	17.1%	19.6%	< 0.001
2 identities	12.5%	11.4%	18.1%	10.5%	17.6%	17.2%	
3 identities	6.6%	5.0%	14.6%	5.7%	12.6%	13.2%	
≥ 4 identities	5.7%	2.7%	21.6%	3.8%	17.4%	17.4%	
Repeated exposure to discrimination							< 0.001
No exposure	53.3%	58.5%	26.8%	59.7%	30.6%	31.1%	
Exposure before COVID-19 only	19.7%	19.1%	21.7%	18.7%	23.3%	21.4%	
Exposure during COVID-19 only	2.6%	2.3%	3.8%	2.2%	4.7%	1.5%	
Repeated exposure	24.4%	20.1%	47.7%	19.5%	41.4%	46.0%	

*Long-term conditions*, long-term conditions that have lasted or are expected to last for six or more months upon the survey (as of August 24, 2020). *LGBTQ2*, lesbian, gay, bisexual, transgender, queer, two-spirit. *Indigenous populations*, First Nations, Métis, or Inuk. *Pre-pandemic*, 2 years before the COVID-19 pandemic; *during pandemic*, January to August (survey recall period), 2020

### Statistical Analyses

Data modeling proceeded in several steps. First, the proportion of each race-migration category was calculated by socio-demographics, chronic conditions, and other covariates. Second, the burden of experiencing identity-based and situation-based discrimination was estimated for each race-migration category. To illustrate comparisons and trajectories of prevalence between the pre-pandemic and during the pandemic period, each domain of discrimination was presented via heatmap visualization. Chi-square tests (*χ*^*2*^) were used to test the statistical differences at the bivariate level for these two steps. Third, multinomial logistic regression models were built to estimate the probability of experiencing cumulative exposure to discrimination (five levels of categories) for each race-migration group compared to CB whites, before and during the COVID-19 pandemic. Two series of models were performed: (1) a crude model that entered only the race-migration nexus and (2) an adjusted model that added all covariates. Finally, another series of binary logistic regression models were fitted to analyze associations between cumulative exposure to discrimination and the probability of having long-term health conditions (yes versus no), while adjusting for all covariates. Statistical analyses were performed using the SPSS software package, Version 26 (IBM Corp., Armonk, NY, USA). To account for multiple testing of a large set of independent variables, a more stringent criterion was employed for the interpretation of *p*-values (*p* < 0.01) for statistical significance, and the 99% confidence intervals (99% CI) were used.

## Results

### Sample Characteristics

Because Canada does not routinely provide statistics by racial groups, it is challenging to compare the ICCED sample to the general population with the same accuracy as in the United States. Table 1 summarizes sample characteristics of key variables and selected covariates stratified by race-migration nexus (all indicators *p*’s < 0.001). The sample (*n* = 32,605) mainly consisted of respondents who were Canadian-born whites (74.9%), female (71.6%), attended university (65.7%), lived in urban areas (89%), and primary spoken English at home (79.9%). Around half of the participants (49.2%) had at least one long-term health condition and one in five participants (20.6%) had two or more conditions. Compared to the majority privileged group — CB whites — Indigenous populations were over-represented in lower educational strata who did not attend university (50% vs 37.8%), while CB racialized minorities were over-represented in younger age strata who aged < 45 years (75.1% vs 48.1%). Indigenous people had the highest proportion reporting barriers in accessing > 1 health services (49.2%), low trust in the health care system (28%), weak sense of community belonging (39.4%), and having a chronic condition (62.7%), especially mental health problems (41.4%) and physical act difficulties (18%). In sharp contrast, white populations, regardless of migration status, reported higher proportions of strong community belonging (around 70%) than racialized communities.

### RQ1: Prevalence of Discrimination Before and During COVID-19

**Fig. 2:**
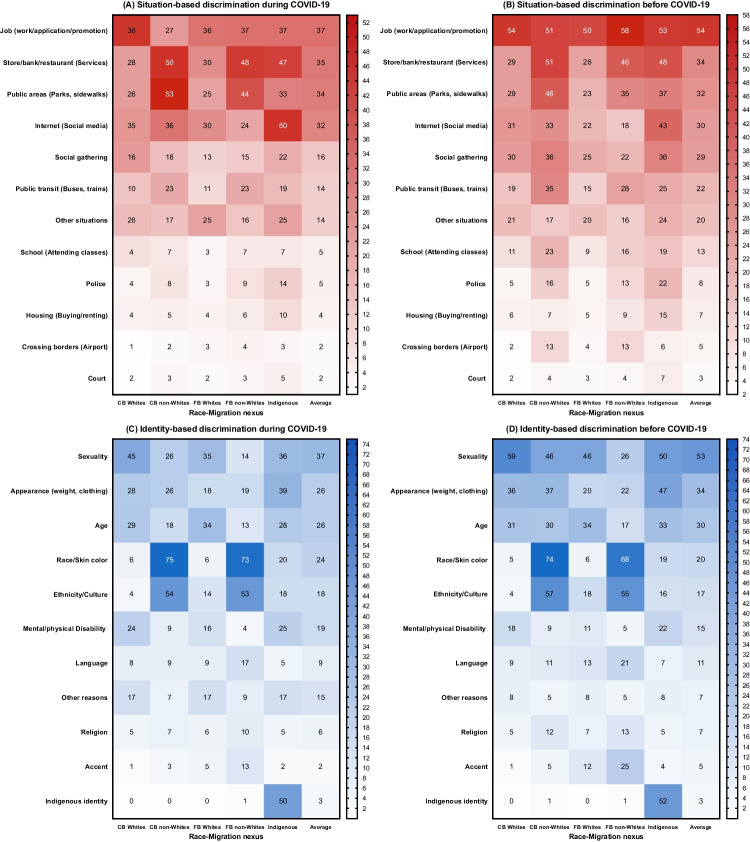
Prevalence of situation- and identity-based discrimination during and before COVID-19 in Canada, stratified by race-migration nexus, ICCED-2020 (*N* = 32,605). Notes: Value in the colored square represents the prevalence of discrimination (darker color indicates a higher percentage — more frequent experience of discrimination; lighter color indicates a lower percentage — less frequent experience of discrimination). The legends for the heatmap are on the right side. CB, Canadian-born; FB, foreign-born (i.e., immigrants)

Among general populations (see Table 1), the overall prevalence of experiencing discrimination substantially reduced during the pandemic than its prevalence at pre-pandemic times (27% vs 44%). This diminishing trend may be due to the fact that many public health measures such as stay-at-home orders and workplace closure were meant to decrease human face-to-face interactions, thus reducing potential discriminatory encounters in public areas. Figure 2A to D show the heat maps to illustrate multi-dimensions of exposure to identity-based and situation-based discrimination across two time periods (see supplement 2 & 3 for more details). For situation-based discrimination (shown in Fig. 2A and B), the diminishing trend was evident by the fact that the prevalence of discrimination (e.g., school setting, workplace, social gathering, public transit) decreased among Canadians during the pandemic, whereas discrimination on the Internet/cyberspace (e.g., social media platform) stood out to be substantially more prevalent than pre-pandemic times (34% vs 30%), especially among Indigenous populations (50% vs 43%). However, notwithstanding this overall diminishing trend of discrimination exposure, racial disparities in experiencing discrimination remain (Fig. 2 A): Compared to CB whites, racialized communities (e.g., CB and FB non-whites) still had higher proportions of perceived situation-based discrimination during the pandemic, especially in these scenarios including being in public recreational/ sidewalk areas (53%, 44% vs 26%), being in public transit (23%, 23% vs 10%), interacting with the police (8%, 9% vs 4%), and crossing the border into Canada (2%, 4% vs 1%).

Regarding identity-based discrimination (shown in Fig. 2C and D), although the prevalence of discrimination stemming from certain marginalized identities (e.g., physical appearance, sexuality, and age) had a downward trend during the COVID-19 times compared to the pre-pandemic period, several contrasting patterns stood out: discrimination stemming from racialization — such as race/skin color (24% vs 20%) and ethnicity/culture (18% vs 17%) — exaggerated during the pandemic relative to pre-pandemic period. In addition, racialized communities consistently reported higher cumulative exposure to discrimination (across all domains of discrimination) across both time periods, whereas white populations consistently reported lower exposure. For example (see Table 1), compared to CB whites, the prevalence of exposure to four or more types of identity-based discrimination was substantially higher among CB non-whites, FB non-whites, and Indigenous populations during the pandemic (9.3%, 8.9%, 9.6% vs 1.3%).

### RQ2: Associations Between Race-Migration Nexus and Perceived Discrimination

**Fig. 3:**
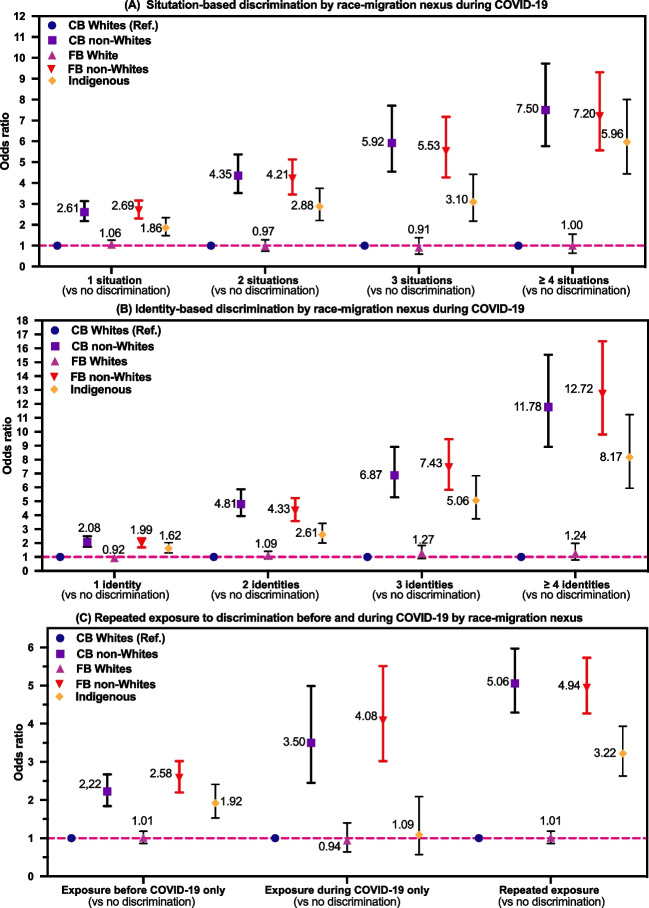
Associations between race-migration nexus and multiple discrimination in Canada, ICCED, August 2020 (*N* = 32,605). Notes: Odds ratios were calculated by multinomial logistic regression fully adjusted by covariates, including age, sex, education, household living arrangement, sexual orientation, marital status, language spoken at home, barriers to health care, and community belonging. To account for multiple testing, a more stringent criterion was adopted: *p* < 0.01 was considered statistically significant, and 99% confidence intervals (99% CI) were used. Given the non-probabilistic nature of the crowdsourcing data in this survey, confidence intervals should be interpreted with extreme caution. CB, Canadian-born; FB, foreign-born (i.e., immigrants)

Figure 3A to C demonstrate the forest plots of associations between the race-migration nexus and exposure to discrimination across two time periods (See supplement 4 for detailed statistics). Findings of both crude and adjusted models suggested that three racialized minoritized groups were at elevated odds of experiencing discrimination than CB whites, and these exposure gaps were not narrowed even during the pandemic times. Interestingly, after full adjustment for socio-demographics, these associations were strengthened (i.e., estimates were enlarged) for racialized immigrants, about the same for CB non-whites, but were slightly attenuated (i.e., estimates were diminished) for the Indigenous population. Most importantly, robust relationships were found between the race-migration nexus and cumulative exposure to discrimination (see Fig. 3 A and B). For example, compared to the dominant CB whites, all racialized communities, including CB non-whites, FB non-whites, and the indigenous population, had a higher likelihood of experiencing both identity-based and situation-based discrimination during the pandemic (*p* < 0.001). In contrast, in the fully adjusted models, FB white was the only group that did not significantly differ from Canadian-born white in terms of discrimination exposure before the COVID-19 pandemic (*p* = 0.205) and during the COVID-19 pandemic (*p* = 0.298).

Compared to CB whites, racialized/immigrant minorities’ likelihood of experiencing discrimination increased, alongside the domains of discrimination being additively intersected (with other social identities/situations) during the pandemic (*p* < 0.001). For example, in the fully adjusted models for situation-based discrimination (Fig. 3 A), while CB racialized minorities were 2.6 times at greater odds than Canadian-born whites to experience discrimination in one situation during the pandemic (OR [odds ratio] = 2.61, 99% CI [confidence interval]: 2.18–3.13), adding one discriminatory situation more than quadruple the likelihood (OR = 4.35, 99% CI 3.52–5.37). The largest jump in odds was observed in the transition from the level of one domain of discrimination to the level of four or more domains of discrimination (OR = 7.50, 99% CI 5.77–9.73). This was also the case for racialized immigrants and Indigenous populations. Similarly, these positive relationships were even stronger among racialized communities with respect to exposure to intersectional identity-based discrimination (Fig. 3 B). Notably, as shown in Fig. 3 C, the likelihood of experiencing repeated exposure to discrimination (i.e., chronic stressor) across two time periods for CB racialized minorities (OR = 5.06, 99% CI 4.29–5.97), racialized immigrants (OR = 4.94, 99% CI 4.27–5.73) and Indigenous populations (OR = 3.22, 99% CI 2.63–3.93) were significantly higher than CB whites, with the exception of FB whites (OR = 1.01, *p* = 0.92).

**Fig. 4:**
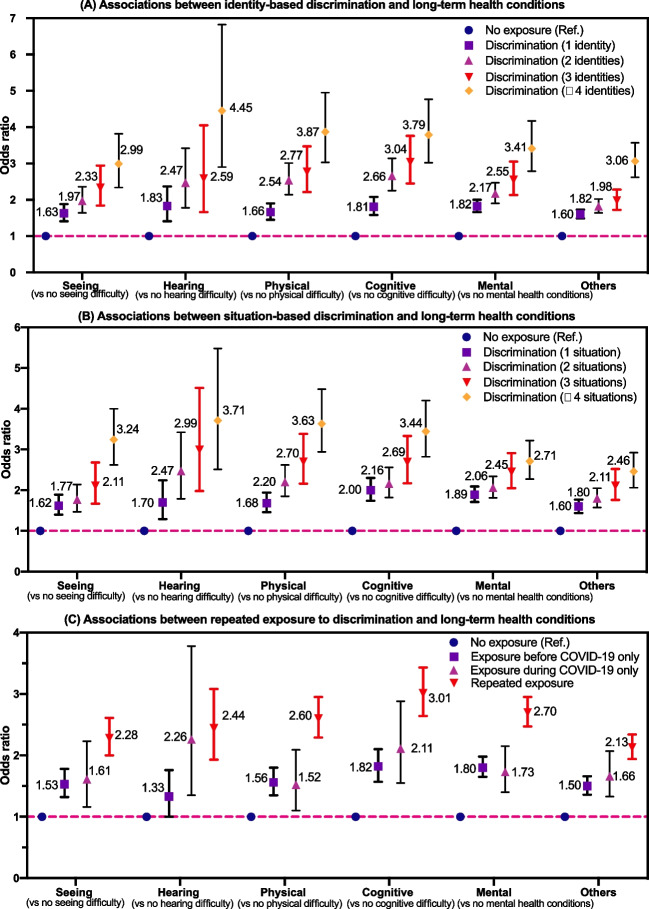
Dose-response relationships between cumulative exposure to discrimination and long-term conditions during COVID-19 in Canada, ICCED, August 2020 (*N* = 32,605). Notes: Odds ratios were calculated by binary logistic regression fully adjusted by covariates, including race-migration nexus, age, sex, education, household living arrangement, sexual orientation, marital status, language spoken at home, barriers to health care, and community belonging. To account for multiple testing, a more stringent criterion was adopted: *p* < 0.01 was considered statistically significant, and 99% confidence intervals (99% CI) were used. During pandemic, January to August 2020 (survey recall period). Given the non-probabilistic nature of the crowdsourcing data in this survey, confidence intervals should be interpreted with extreme caution

Interestingly, in the context of COVID-19 pandemic (Fig. 3 C), CB racialized respondents were more than three times as likely (OR = 3.50, 99% CI 2.45–4.99), and FB racialized respondents were more than four times as likely (OR = 4.08, 99% CI 3.02–5.51) to report new experiences of discrimination, whereas FB whites and Indigenous populations did not differ from CB whites in terms of becoming new victims of discrimination triggered by the pandemic. Lastly, when it comes to the comparison between situation- and identity-based discrimination (Fig. 3 A and B), one contrasting pattern stood out: compared to CB whites, higher ORs were reported among CB racialized respondents (ORs 5.92 to 7.50) than FB racialized respondents (ORs 5.53 to 7.20) for ≥ 3 situation-based discrimination; On the opposite, higher ORs were reported among FB racialized respondents (ORs 7.43 to 12.72) than CB racialized respondents (ORs 6.87 to 11.78) for ≥ 3 identity-based discrimination.

### RQ3: Associations Between Perceived Discrimination and Long-Term Conditions

Figure 4 A to C present the forest plots of the binary logistic regression model for estimating the likelihood of long-term health conditions by cumulative exposure to discrimination (see supplement 5 for detailed statistics). Results of fully adjusted models suggest that exposure to discrimination, regardless of any domain or any period, is robustly associated with long-term conditions via dose-response patterns. Notably, salient gradient effects of discrimination on long-term conditions stood out consistently for six health outcomes measured in the ICCED. For instance, during the pandemic, with increasing levels of exposure to identity-based discrimination (see Fig. 4 A), the odds of reporting physical impairment significantly escalated, ranging from victims of one form of discrimination with 68% higher odds (OR = 1.66, 99% CI 1.44–1.90) to victims of four forms of discrimination having close to quadruple the odds (OR = 3.87, 99% CI 3.03–4.95), compared to non-victims. Likewise, for situation-based discrimination (see Fig. 4 B), as the discrimination level increased, the proportion of respondents who reported having mental health conditions increased gradually (ORs range from 1.89 to 2.71), among whom the odds of experiencing mental health-related difficulty in the group in the highest category of exposure to discrimination almost triple (OR = 2.71, 99% CI 2.27–3.22) in comparison to non-victims. Lastly, as shown in Fig. 4 C, the odds of having six long-term conditions among victims with exposure to discrimination in only a single period (either pre-pandemic or during the pandemic) were significantly higher (ORs range from 1.33 to 2.26), compared to non-victims, being exposure to chronic discrimination (i.e., having repeated exposure to discrimination across two time periods) further increased such odds (ORs range from 2.13 to 3.01)

## Discussion

 This nationwide study investigates the associations between race-migration nexus, accumulation of multiple forms of perceived discrimination, and its mental and physical health consequences during the COVID-19 pandemic in Canada. These relationships may shed some light on who may have higher susceptibility to negative sequelae of COVID-19 severe illness. The findings reveal that social status–based discrimination disproportionately affects racialized and immigrant minorities, and there is a strong linkage between accumulation of discrimination and adverse health outcomes. Although Canadians share a publicly funded “universal” health care system with free medical services provided at the point of first contact [26, 27], Canada is not immune to structural drivers of health disparities [36, 37].

The present study offers three critical contributions. First, a cumulative relationship between experiences of intersectional discrimination and the race-migration nexus was quantified in Canada. Canadian-born racialized people, racialized immigrants, and the Indigenous population were more likely to be victims of multiple forms of discrimination, whereas white immigrants were not statistically different than CB whites on most occasions. This finding suggests that the use of “migration status” as a monolithic group was problematic in that it would underestimate discrimination for racialized immigrants [26, 27]. In fact, racialized people with transnational backgrounds are more prone to dual oppression, supporting the “double Jeopardy” theory [27, 38]. These stressors could stem from the devaluation of foreign credentials, overrepresentation in low-wage precarious occupations, and concentration in deprived neighborhoods [39], all of which may counter the “healthy immigrant effect” as immigrants’ residence time lengthened. The magnitude of Indigenous-white disparities in exposure to discrimination also highlights the repercussion of colonization on First Nations communities.

Furthermore, the gradient effect of exposure to multiple discrimination among racialized people observed in the present study is novel because it capture the additive burden of multiple and coexisting forms of unfair treatments that may occur on the ground of more than one stigmatized characteristic or one occasion of living conditions. These findings acknowledge the concept of sequential multiple discrimination that may give rise to “cumulative disadvantage” and extend the existing literature that overwhelmingly focused on a single form of discrimination. Growing evidence on discrimination attributions in the U.S. has documented that [40, 41], compared to no/minimal discrimination group, membership in the high discrimination attributions class (i.e., attributed discrimination to multiple reasons/sources), generated by the latent class analysis, was associated with poor self-rated health [41], depressive symptoms [40, 42], and greater all-cause mortality risk [43].

Second, the findings of this study are particularly relevant in the context of the COVID-19 pandemic, where both CB and FB racialized populations tripled the odds of becoming new victims of discrimination during these turbulent times. This evidence echoes to fact that racialized individuals have become more common to encounter hostile interactions and racist views since the pandemic began. This study further revealed the concerning magnitude of discrimination by documenting that almost one-in-two racialized Canadians have experienced discrimination amid the COVID-19 outbreak. Previous studies have documented that racialized people were structurally placed in disadvantaged social positions with increased risk of exposure to pathogens [44], such as precarious work which requires extensive interaction with the public (e.g., retail positions, cleaners, or cashiers) [30], the need to use public transit rather than driving a car [45], and living in ov ercrowded housing that makes self-isolation difficult [30]. Racialized populations are also over-represented in frontline health care occupations including personal support worker and nursing [3]. Black and Indigenous populations in Canada are more prone to everyday discrimination associated with chronic conditions [37]. In fact, in the digital era, the practices of othering and the longstanding negative stereotypes targeting racialized and immigrant minorities, particularly Asian and Chinese communities, have been further reinforced in both online and offline worlds during the COVID-19 crisis [8], such as the unjustifiable and xenophobic narrative of “China virus” or “Wuhan virus”. Interestingly, this study also found that, when compared to CB whites, the odds of reporting situation-based discrimination were higher among CB racialized than FB racialized groups, while that of identity-based was the opposite. It may be that native-born racialized individuals are more sensitive to discriminatory interactions in daily situations, resulting from early life socialization experiences (e.g., education and schooling) that lead to their heightened expectations of social inclusion as well as the acquisition of language, habitus, and values in the host society during formative developmental years [46].

Third, this study broadens the literature on the discrimination-health relationship by documenting that both intersectional discrimination (in terms of multiple forms) and chronic discrimination (in terms of duration) were associated with poor mental and physical health outcomes via dose-response patterns among general populations in Canada. Analyses of these categories were consistent with the notion that perceived discrimination represents important psychosocial stressors [47] and the biological consequences of exposure to toxic stress [10]. Stress accumulation through exposure to various forms of discrimination, stereotypes, and microaggression constitutes crucial pathways through which social inequalities and adversities could get “under the skin.” Previous research has demonstrated the individual-level etiologic mechanism to explain how persistent social inequalities and discrimination related to living conditions and work environments could be embodied to undermine biological regulatory systems [48]. For example, minoritized individuals (i.e., stigmatized groups) on the receiving end of prolonged discrimination are often in a state of hypervigilance (e.g., heightened watchfulness). Such experience of chronic stress may over-activate individuals’ stress response pathways for an extended period [49, 50], which can have a wear-and-tear effect on the body over time by increasing the allostatic load [50] — a measure of multiple physiologic parameters such as blood pressure, daily cortisol variations, and cholesterol levels [51] — resulting in a wide range of stress-related diseases [51–53] and even biological alteration that accelerates epigenetic aging [50]. The experience of discrimination can also lead to stress-related behavioral changes that are health-damaging, including increased intake of alcohol, tobacco, and other substance use [50, 54]. As highlighted in a recent Lancet paper, because "the importance for health of biological responses to discrimination has been severely under-recognised" [50], this study provides additional evidence to support the gradient effect of discrimination-health relationship.    

Lastly, it is also worth mentioning that even with a public-funded health care system, previous Canadian studies have found that racialized and immigrant minorities may face a myriad of barriers to accessing health care services [4, 55], arising from structural discrimination, such as language obstacle [56], misinterpretation of symptoms in the cross-cultural clinical setting [57], structural labor shortage of cultural-responsive family doctors [58, 59] and medical mistrust [60]. A recent population-based study found that compared to the CB whites, racialized immigrant older adults were more likely to have multimorbidity, lack a usual source of care, and report unmet care needs for chronic conditions due to greater acceptability barriers, suggesting an impact of discrimination at the societal level [26]. Consistently, another related study revealed that racialized immigrants with mental disorders tended to underutilize mental health services as evidenced by lower odds of past-year mental health consultations than their CB white peers with mental disorders [27]. Although the ICCED did not capture exposure to discrimination in the health care setting, our findings indicate that Indigenous people had the highest proportion reporting barriers in accessing two or more health services (49.2% vs 36.5%) and having low trust in the Canadian health care system (28% vs 12%), compared to the dominant CB whites. Such inequalities in health care services in Canada still posed significant threat to the achievement of SDGs and may further exacerbate health inequities in long-term health conditions faced by racialized and minority communities [4, 55, 60].

## Limitations

First, the ICCED did not specifically ask respondents about exposure to discrimination for certain social identities, especially social class and migration status, and thus, the survey could not capture structural oppression associated with classism, nationalism, and xenophobia. Also, the perceived discrimination measured in this study may serve as a global discrimination indicator that may only capture major, stressful life events (e.g., the target of overt acts of bias) that remain imprinted in respondents’ memories [46]. Those subtle, minor discriminatory encounters (e.g., microaggressions) would be better captured by the well-established Everyday Discrimination Scale [61], which was once available in Statistics Canada’s 2013 Canadian Community Health Survey rapid response module. It would also be beneficial to compare these findings to the General Social Survey – Social Identity (2020 circle) which captured a similar component of the discrimination experiences (5 year before and since the pandemic). Second, Statistics Canada’s Crowdsourcing Data series did not employ a probabilistic sampling technique. Therefore, results in this study are not generalizable to the general Canadian population despite benchmarking techniques. The online survey method of this data further excludes individuals who did not have access to the Internet or digital devices and is subject to potential sampling bias. For instance, compared to the CB whites, racialized minorities, both Canadian-born and foreign-born, were still over-represented at younger age of the cohort (< 45 years old) in this sample (75%, 57% vs 48%). This uneven age distribution across race-migration nexus is problematic when it comes to the investigation of chronic condition distribution, with racialized groups being much younger than the white population in this sample. Since age cohorts of this sample were so imbalanced by the race-migration nexus and with age coded as a binary variable (45 years below or above) instead of age in years, it would be misleading to compare disparities of long-term health conditions by racial and immigrant groups even adjusted for age in multivariable regression model.

Moreover, the data are cross-sectional in nature, prohibiting results from causal inference. As such, the findings should be interpreted more as correlation, rather than predictive or “consequences” per se. For example, one reason for identity-based discrimination is mental/physical disability, and this item may overlap with long-term conditions (as outcome variables in the present study) that may give rise to discrimination. Furthermore, considering the chronicity of long-term conditions in this study, people with disadvantaged positions at the intersection of race and migration (e.g., CB non-whites, Indigenous populations) may be at higher risk of living with these conditions over the life course, and thus, they are more likely to require additional health-care resources and access to coordinated care in chronic illness management in later life. Future studies could adopt a longitudinal approach in examining whether barriers in health care access at the baseline may lead to a greater burden of chronic illness at a later stage among racialized and immigrant minorities. In addition, the measure of pre-pandemic discrimination (2 years prior to the COVID-19 pandemic) was retrospectively based on respondents’ recall memory and the responses may involve recall bias. Many individuals with medical conditions may have not been diagnosed. Such a limitation of under-reporting would probably bias the results toward the null and result in an underestimate of the population at risk of severe COVID-19 illness. Lastly, the complexity of social makers (e.g., age, gender, income, education) could potentially exacerbate inequities, and future research could investigate how the race-migration nexus intersects with other SDoH to shape social patterning of population health and disease risk in Canada.

## Conclusion

Despite Canada has a universal health system, this study has demonstrated inequalities by race-migration nexus in the prevalence of accumulative exposure to discrimination associated with chronic health conditions over a substantial period of time (two year before and during the COVID-19 pandemic). The scientific evidence from this study underscores that combating “root causes” of racial and migration-based health inequalities — multiple forms of discrimination on the grounds of race/ethnicity, indigeneity, language, and religion, should be placed at the center of public health interventions to protect all humanity from pandemic threats anywhere and at any time. [62]. These observed racial migration inequities in exposure to discrimination and associated adverse health outcomes are symptoms (as well as warning indicators) of upstream systemic inequity that may significantly undermine community engagement, trust building and public health promotion in the Canadian society. Since Canada has far lagged behind the United States in systemically collecting race-based data on discrimination, future collective efforts should enhance data collection and surveillance system to investigate the heterogeneity and intragroup differences of racialized immigrant communities, such as country of origin, admission class, and disaggregated racial/ethnic composition. In all countries – whether low-, middle- or high-income –reducing health inequalities is one of the key ingredients to achieve the United Nation’s 2030 SGDs Agenda. To create equitable solutions in addressing infectious disease threat and future outbreak, this study highlights that pandemic responses should be re-designed to include anti-discrimination policies to tackle upstream social inequities, particularly empowering racialized and immigrant individuals to cope with day-to-day life challenges during unprecedented public health emergency.

## Supplementary Information

Below is the link to the electronic supplementary material.

Supplementary file1 (DOCX 103 KB)

## Data Availability

The public use microdata file of the Crowdsourcing survey is available to Canadian researchers via Statistics Canada’s Data Liberation Initiative and to international researchers by request at dli-idd@statcan.gc.ca from Statistics Canada.

## Declarations

### Disclaimer

The opinions expressed in this manuscript are the author’s own and do not reflect the views of Statistics Canada.

## References

[1] Abrams EM, Szefler SJ. COVID-19 and the impact of social determinants of health. Lancet Respir Med. 2020;8(7):659–61. 10.1016/S2213-2600(20)30234-4. 32437646 10.1016/S2213-2600(20)30234-4 PMC7234789

[2] Bambra C, Riordan R, Ford J, Matthews F. The COVID-19 pandemic and health inequalities. J Epidemiol Community Health. 2020;74(11):964–8. 10.1136/jech-2020-214401. 32535550 10.1136/jech-2020-214401 PMC7298201

[3] Wright L, Steptoe A, Fancourt D. Are we all in this together? longitudinal assessment of cumulative adversities by socioeconomic position in the first 3 weeks of lockdown in the UK. J Epidemiol Community Health. 2020. 10.1136/jech-2020-214475. 32503892 10.1136/jech-2020-214475 PMC7298206

[4] Lin SL. Access to health care among racialised immigrants to Canada in later life: a theoretical and empirical synthesis. Ageing Soc. 2022;42(8):1735–59.

[5] Marmot M. Social determinants of health inequalities. Lancet. 2005;365(9464):1099–104. 10.1016/S0140-6736(05)71146-6. 15781105 10.1016/S0140-6736(05)71146-6

[6] Liu Y, Finch BK, Brenneke SG, Thomas K, Le PD. Perceived discrimination and mental distress amid the COVID-19 pandemic: evidence from the understanding America study. Am J Prev Med. 2020;59(4):481–92. 10.1016/j.amepre.2020.06.007. 32829968 10.1016/j.amepre.2020.06.007 PMC7336127

[7] Yashadhana A, Derbas A, Biles J, Grant J. Pandemic-related racial discrimination and its health impact among non-indigenous racially minoritized peoples in high-income contexts: a systematic review. Health Promot Int. 2022;37(2):daab144. 10.1093/heapro/daab144. 34595531 10.1093/heapro/daab144 PMC8500046

[8] Strassle PD, Stewart AL, Quintero SM, et al. COVID-19-related discrimination among racial/ethnic minorities and other marginalized communities in the United States. Am J Public Health. 2022;112(3):453–66. 10.2105/AJPH.2021.306594. 35196054 10.2105/AJPH.2021.306594 PMC8887166

[9] Lin S. COVID-19 pandemic and im/migrants’ elevated health concerns in Canada: vaccine hesitancy, anticipated stigma, and risk perception of accessing care. J Immigr Minor Health. 2022;24(4):896–908. 10.1007/s10903-022-01337-5. 35212825 10.1007/s10903-022-01337-5 PMC8874751

[10] Devakumar D, Shannon G, Bhopal SS, Abubakar I. Racism and discrimination in COVID-19 responses. Lancet. 2020;395(10231):1194. 10.1016/S0140-6736(20)30792-3. 32246915 10.1016/S0140-6736(20)30792-3 PMC7146645

[11] Lin SL. The “loneliness epidemic”, intersecting risk factors and relations to mental health help-seeking: a population-based study during COVID-19 lockdown in Canada. J Affect Disord. 2023;320:7–17. 36058359 10.1016/j.jad.2022.08.131 PMC9436782

[12] Bailey ZD, Krieger N, Agénor M, Graves J, Linos N, Bassett MT. Structural racism and health inequities in the USA: evidence and interventions. Lancet. 2017;389(10077):1453–63. 28402827 10.1016/S0140-6736(17)30569-X

[13] Krieger N. Discrimination and health inequities. Int J Health Serv. 2014;44(4):643–710. 25626224 10.2190/HS.44.4.b

[14] Krieger N. Embodying inequality: a review of concepts, measures, and methods for studying health consequences of discrimination. Int J Health Serv. 1999;29(2):295–352. 10.2190/M11W-VWXE-KQM9-G97Q. 10379455 10.2190/M11W-VWXE-KQM9-G97Q

[15] Krieger N. Embodiment: a conceptual glossary for epidemiology. J Epidemiol Community Health. 2005;59(5):350–5. 10.1136/jech.2004.024562. 15831681 10.1136/jech.2004.024562 PMC1733093

[16] Link BG, Phelan J. Social conditions as fundamental causes of disease. J Health Soc Behav. 1995;80–94. 10.2307/2626958. 7560851

[17] Palmer RC, Ismond D, Rodriquez EJ, Kaufman JS. Social determinants of health: future directions for health disparities research. Am J Public Health. 2019;109(S1):S70–1. 10.2105/AJPH.2019.304964. 30699027 10.2105/AJPH.2019.304964 PMC6356128

[18] Petrilli CM, Jones SA, Yang J, et al. Factors associated with hospital admission and critical illness among 5279 people with coronavirus disease 2019 in New York City: prospective cohort study. BMJ. 2020;369:m1966. 10.1136/bmj.m1966. 32444366 10.1136/bmj.m1966 PMC7243801

[19] Brown TH. Racial stratification, immigration, and health inequality: a life course-intersectional approach. Soc Forces. 2018;96(4):1507–40. 10.1093/sf/soy013.

[20] Gkiouleka A, Huijts T, Beckfield J, Bambra C. Understanding the micro and macro politics of health: inequalities, intersectionality & institutions - a research agenda. Soc Sci Med. 2018;200:92–8. 10.1016/j.socscimed.2018.01.025. 29421476 10.1016/j.socscimed.2018.01.025

[21] Crenshaw K. Mapping the margins: intersectionality, identity politics, and violence against women of color. Stanford Law Rev. 1990;43:1241. 10.2307/1229039.

[22] Earnshaw VA, Rosenthal L, Gilstad-Hayden K, Carroll-Scott A, Kershaw TS, Santilli A, Ickovics JR. Intersectional experiences of discrimination in a low-resource urban community: an exploratory latent class analysis. J Community Appl Soc Psychol. 2018;28(2):80–93.

[23] Balsam KF, Molina Y, Beadnell B, Simoni J, Walters K. Measuring multiple minority stress: the LGBT People of Color Microaggressions Scale. Cult Divers Ethn Minor Psychol. 2011;17(2):163. 10.1037/a0023244 PMC4059824 21604840

[24] Cuddy AJ, Fiske ST, Glick P. Warmth and competence as universal dimensions of social perception: the stereotype content model and the BIAS map. Adv Exp Soc Psychol. 2008;40:61–149.

[25] Scheim AI, Bauer GR. The Intersectional Discrimination Index: development and validation of measures of self-reported enacted and anticipated discrimination for intercategorical analysis. Soc Sci Med. 2019;226:225–35. 30674436 10.1016/j.socscimed.2018.12.016

[26] Lamson Lin S, Fang L. Chronic care for all? the intersecting roles of race and immigration in shaping multimorbidity, primary care coordination, and unmet health care needs among older Canadians. J Gerontol: Ser B. 2022;78:302–18. 10.1093/geronb/gbac125 36044754

[27] Lamson LS. Inequities in mental health care facing racialized immigrant older adults with mental disorders despite universal coverage: a population-based study in Canada. J Gerontol: Ser B. 2023;78(9):1555–71. 10.1093/geronb/gbad036. 10.1093/geronb/gbad036 PMC10461535 36842070

[28] Ferrer I, Grenier A, Brotman S, Koehn S. Understanding the experiences of racialized older people through an intersectional life course perspective. J Aging Stud. 2017;41:10–7. 10.1016/j.jaging.2017.02.001. 28610750 10.1016/j.jaging.2017.02.001

[29] Tuyisenge G, Goldenberg SM. COVID-19, structural racism, and migrant health in Canada. The Lancet. 2021;397(10275):650–2. 10.1016/S0140-6736(21)00215-4. 10.1016/S0140-6736(21)00215-4 PMC9755558 33539727

[30] McGrail K, Morgan J, Siddiqi A. Looking back and moving forward: addressing health inequities after COVID-19. Lancet Reg Health Am. 2022;9. 10.1016/j.lana.2022.100232. 10.1016/j.lana.2022.100232 PMC8928332 35313508

[31] Khan MM, Kobayashi K, Vang ZM, Lee SM. Are visible minorities “invisible” in Canadian health data and research? A scoping review. Int J Migr Health Soc Care. 2017;13(1):126–43.

[32] Thomason ME, Hendrix CL, Werchan D, Brito NH. Perceived discrimination as a modifier of health, disease, and medicine: empirical data from the COVID-19 pandemic. Transl Psychiatry. 2022;12(1):284. 35840584 10.1038/s41398-022-02047-0 PMC9285192

[33] Sáenz R, Manges DK. A call for the racialization of immigration studies: on the transition of ethnic immigrants to racialized immigrants. Soc Race Ethn. 2015;1(1):166–80. 10.1177/2332649214559287.

[34] Erel U, Murji K, Nahaboo Z. Understanding the contemporary race–migration nexus. Ethn Racial Stud. 2016;39(8):1339–60. 10.1080/01419870.2016.1161808.

[35] Government of Canada. Crowdsourcing: impacts of COVID-19 on Canadians’ experiences of discrimination public use microdata file. 2020; 10.25318/45250008-eng.

[36] Etowa J, Sano Y, Hyman I, et al. Difficulties accessing health care services during the COVID-19 pandemic in Canada: examining the intersectionality between immigrant status and visible minority status. Int J Equity Health. 2021;20:255. 10.1186/s12939-021-01593-1. 34915891 10.1186/s12939-021-01593-1 PMC8674863

[37] Wallace S, Nazroo J, Bécares L. Cumulative effect of racial discrimination on the mental health of ethnic minorities in the United Kingdom. Am J Public Health. 2016;106(7):1294–300. 27077347 10.2105/AJPH.2016.303121 PMC4984732

[38] Ramraj C, Shahidi FV, Darity W, Kawachi I, Zuberi D, Siddiqi A. Equally inequitable? A cross-national comparative study of racial health inequalities in the United States and Canada. Soc Sci Med. 2016;161:19–26. 10.1016/j.socscimed.2016.05.028. 27239704 10.1016/j.socscimed.2016.05.028

[39] Siddiqi A, Shahidi FV, Ramraj C, Williams DR. Associations between race, discrimination and risk for chronic disease in a population-based sample from Canada. Soc Sci Med. 2017;194:135–41. 10.1016/j.socscimed.2017.10.009. 29100138 10.1016/j.socscimed.2017.10.009

[40] Erving CL, Cobb RJ, Sheehan C. Attributions for everyday discrimination and all-cause mortality risk among older Black women: a latent class analysis approach. Gerontologist. 2023;63(5):887–99. 10.1093/geront/gnac080. 35678164 10.1093/geront/gnac080 PMC10268592

[41] Lu P, Kong D, Shelley M, Davitt JK. Intersectional discrimination attributions and health outcomes among American older adults: a latent class analysis. Int J Aging Hum Dev. 2022;95(3):267–85. 10.1177/00914150211066560. 34931874 10.1177/00914150211066560

[42] Cobb RJ, Sheehan CM, Louie P, Erving CL. Multiple reasons for perceived everyday discrimination and all-cause mortality risk among older Black adults. J Gerontol A Biol Sci Med Sci. 2022;77(2):310–4. 10.1093/gerona/glab281. 34605539 10.1093/gerona/glab281 PMC9012980

[43] Cobb RJ, Rodriguez VJ, Brown TH, et al. Attribution for everyday discrimination typologies and mortality risk among older black adults: evidence from the health and retirement study? Soc Sci Med. 2023;316:115166. 10.1016/j.socscimed.2022.115166. 36450613 10.1016/j.socscimed.2022.115166 PMC13321940

[44] Lum TY, Vanderaa JP. Health disparities among immigrant and non-immigrant elders: the association of acculturation and education. J Immigr Minor Health. 2010;12(5):743–53. 19184599 10.1007/s10903-008-9225-4

[45] Edge S, Newbold B. Discrimination and the health of immigrants and refugees: exploring Canada’s evidence base and directions for future research in newcomer receiving countries. J Immigr Minor Health. 2013;15(1):141–8. 22729289 10.1007/s10903-012-9640-4

[46] Vang ZM, Chang Y. Immigrants’ experiences of everyday discrimination in Canada: unpacking the contributions of assimilation, race, and early socialization. Int Migr Rev. 2019;53(2):602–31. 10.1177/0197918318764871.

[47] Bavel JJV, Baicker K, Boggio PS, et al. Using social and behavioural science to support COVID-19 pandemic response. Nat Hum Behav 2020;4(5):460–471. https://search.proquest.com/docview/2404626527. 10.1038/s41562-020-0884-z. 10.1038/s41562-020-0884-z 32355299

[48] Raphael D, Curry-Stevens A, Bryant T. Barriers to addressing the social determinants of health: insights from the Canadian experience. Health Policy. 2008;88(2–3):222–35. 10.1016/j.healthpol.2008.03.015. 18471923 10.1016/j.healthpol.2008.03.015

[49] Gee GC, Spencer MS, Chen J, Takeuchi D. A nationwide study of discrimination and chronic health conditions among Asian Americans. Am J Public Health. 2007;97(7):1275–82. 10.2105/AJPH.2006.091827. 17538055 10.2105/AJPH.2006.091827 PMC1913081

[50] Selvarajah S, Corona Maioli S, Deivanayagam TA, et al. Racism, xenophobia, and discrimination: mapping pathways to health outcomes. Lancet. 2022;400(10368):2109–24. 10.1016/S0140-6736(22)02484-9. 36502849 10.1016/S0140-6736(22)02484-9

[51] Seeman T, Epel E, Gruenewald T, Karlamangla A, McEwen BS. Socio-economic differentials in peripheral biology: cumulative allostatic load. Ann N Y Acad Sci. 2010;1186:223–39. 10.1111/j.1749-6632.2009.05341.x. 20201875 10.1111/j.1749-6632.2009.05341.x

[52] Geronimus AT, Hicken M, Keene D, Bound J. “Weathering” and age patterns of allostatic load scores among blacks and whites in the United States. Am J Public Health. 2006;96(5):826–33. 10.2105/AJPH.2004.060749. 16380565 10.2105/AJPH.2004.060749 PMC1470581

[53] James SA, Van Hoewyk J, Belli RF, Strogatz DS, Williams DR, Raghunathan TE. Life-course socioeconomic position and hypertension in African American men: the Pitt County study. Am J Public Health. 2006;96(5):812–7. 10.2105/AJPH.2005.076158. 16571689 10.2105/AJPH.2005.076158 PMC1470586

[54] Riley TN, Clifton RL, Khazvand S, Zapolski TCB. Discrimination and substance use: examining the moderating role of impulsivity among racial-ethnic minority adolescents. Subst Use Misuse. 2021;56(6):897–904. 10.1080/10826084.2021.1899235. 33759684 10.1080/10826084.2021.1899235 PMC8868491

[55] Lin SL. Healthy ageing in a foreign land? Examining health care inequities faced by older racialized immigrants in the Canadian community health survey (2015–2018)(Publication No. 29215851) [Doctoral dissertation, University of Toronto]. ProQuest Dissertations & Thesis A&I. (ID: 2736292777). 2022. https://hdl.handle.net/1807/128348.

[56] Lin S, Kobayashi K, Tong H, Davison KM, Arora SR, Fuller-Thomson E. Close relations matter: the association between depression and refugee status in the Canadian Longitudinal Study on Aging (CLSA). J Immigr Minor Health. 2020;22(5):946–56. 31974926 10.1007/s10903-020-00980-0

[57] Lin S. Healthy immigrant effect or under-detection? examining undiagnosed and unrecognized late-life depression for racialized immigrants and nonimmigrants in Canada. J Gerontol Ser B. 2023;79(3):gbad104. 10.1093/geronb/gbad104 PMC11036341 37498769

[58] Farid D, Li P, Da Costa D, Afif W, Szabo J, Dasgupta K, Rahme E. Undiagnosed depression, persistent depressive symptoms and seeking mental health care: analysis of immigrant and non-immigrant participants of the Canadian Longitudinal Study of Aging. Epidemiol Psychiatr Sci. 2020;29:e158. 32792036 10.1017/S2045796020000670 PMC7443777

[59] Campbell-Scherer D, Chiu Y, Ofosu NN, Luig T, Hunter KH, Jabbour B, et al. Illuminating and mitigating the evolving impacts of COVID-19 on ethnocultural communities: a participatory action mixed-methods study. CMAJ. 2021;193(31):E1203–12. 10.1503/cmaj.210131. 34373268 10.1503/cmaj.210131 PMC8367425

[60] Kangmennaang J, Siiba A, Bisung E. Does trust mediate the relationship between experiences of discrimination and health care access and utilization among minoritized Canadians during COVID-19 pandemic? J Racial Ethn Health Disparities. 2023;1–11. 10.1007/s40615-023-01809-w. 10.1007/s40615-023-01809-w 37787945

[61] Williams DR, Yu Y, Jackson JS, Anderson NB. Racial differences in physical and mental health: socio-economic status, stress and discrimination. J Health Psychol. 1997;2(3):335–51. 10.1177/135910539700200305. 22013026 10.1177/135910539700200305

[62] Sylvia Chou WY, Gaysynsky A. Racism and xenophobia in a pandemic: interactions of online and offline worlds. Am J Public Health. 2021;111(5):773–5. 33826388 10.2105/AJPH.2021.306230 PMC8033987

