# Correction: Immigrant and Racialized Populations’ Cumulative Exposure to Discrimination and Associations with Long-Term Conditions During COVID-19: A Nationwide Large-Scale Study in Canada

**DOI:** 10.1007/s40615-024-02111-z

**Published:** 2024-08-01

**Authors:** Shen (Lamson) Lin

**Affiliations:** 1 Department of Social and Behavioural Sciences, City University of Hong Kong, Hong Kong SAR, China; 2 Factor-Inwentash Faculty of Social Work, University of Toronto, Toronto, Canada; 3 Oxford Institute of Population Ageing, University of Oxford, Oxford, UK

**Correction: Journal of Racial and Ethnic Health Disparities**

10.1007/s40615-024-02074-1

Due to an error during production, the graphic for Fig. 2B was incomplete in the article as originally published. The publisher apologies for the error.

The original article has been corrected.

