# Supplementary material for: Immigrant and Racialized Populations’ Cumulative Exposure to Discrimination and Associations with Long-Term Conditions During COVID-19: A Nationwide Large-Scale Study in Canada
Source: J Racial Ethn Health Disparities. 2024 Jul 17;12(4):2607–22. doi: 10.1007/s40615-024-02074-1 (PMC12241279; doi:10.1007/s40615-024-02074-1)
Supplement: Supplementary file 1 — Supplementary file1 (DOCX 103 KB) [file 40615_2024_2074_MOESM1_ESM.docx]

**Appendix:**

**Supplemental Material (Online Only)**

**Supplement 1. Survey Questions for Key Measures from ICCED-2020 by Statistics Canada**

| **Discrimination** | The next questions are about your experience with discrimination before and during the COVID-19 pandemic. | |
| --- | --- | --- |
| **Identity-based discrimination (pre-pandemic)** | In the 2 years before the COVID-19 pandemic, have you experienced discrimination or been treated unfairly by others in Canada because of any of the following? | ON-SCREEN HELP: Select all that apply.   1. Your Indigenous identity 2. Your ethnicity or culture 3. Your race or skin colour 4. Your religion 5. Your language 6. Your accent 7. Your physical appearance (*Help text: Include discrimination on the basis of weight, height, hair style or colour, clothing, jewelry, tattoos and other physical characteristics. Exclude discrimination on the basis of skin colour*). 8. Your sex *(Help text: Sex refers to sex assigned at birth)* 9. Your sexual orientation (*Help text: e.g., heterosexual, lesbian, gay, bisexual*) 10. Your gender identity or expression (*Help text: Include gender diverse identities such as two-spirit or non- binary*). 11. Your age 12. A physical or mental disability 13. Some other reason 14. Did not experience discrimination   (Don't know, Refusal not allowed) |
| **Identity-based discrimination (pandemic)** | Since the beginning of the COVID-19 pandemic, have you experienced discrimination or been treated unfairly by others in Canada because of any of the following? | ON-SCREEN HELP: Select all that apply.   1. Your Indigenous identity 2. Your ethnicity or culture 3. Your race or skin colour 4. Your religion 5. Your language 6. Your accent 7. Your physical appearance (*Help text: Include discrimination on the basis of weight, height, hair style or colour, clothing, jewelry, tattoos and other physical characteristics. Exclude discrimination on the basis of skin colour*). 8. Your sex *(Help text: Sex refers to sex assigned at birth)* 9. Your sexual orientation (*Help text: e.g., heterosexual, lesbian, gay, bisexual*) 10. Your gender identity or expression (*Help text: Include gender diverse identities such as two-spirit or non- binary*). 11. Your age 12. A physical or mental disability 13. Some other reason 14. Did not experience discrimination   (Don't know, Refusal not allowed) |
| **Situation-based discrimination (pre-pandemic)** | In the 2 years before the COVID-19 pandemic, in **what types of situations** have you experienced discrimination or been treated unfairly by others in Canada? | ON-SCREEN HELP: Select all that apply.   1. In a store, bank or restaurant 2. When attending school or classes 3. On the internet, including social media platforms 4. At work or when applying for a job or promotion 5. When seeking or applying for housing (Help text: e.g., buying or renting) 6. When interacting with the police 7. When interacting with the courts 8. When crossing the border into Canada (Help text: Exclude incidences of discrimination upon leaving Canada.) 9. While attending social gatherings 10. While using public areas, such as parks and sidewalks 11. While using public transit, such as buses, trains, or taxis 12. Any other situation   (Don't know, Refusal not allowed) |
| **Situation-based discrimination (pandemic)** | Since the beginning of the COVID-19 pandemic, in **what types of situations** have you experienced discrimination or been treated unfairly by others in Canada? | ON-SCREEN HELP: Select all that apply.   1. In a store, bank or restaurant 2. When attending school or classes 3. On the internet, including social media platforms 4. At work or when applying for a job or promotion 5. When seeking or applying for housing (Help text: e.g., buying or renting) 6. When interacting with the police 7. When interacting with the courts 8. When crossing the border into Canada (Help text: Exclude incidences of discrimination upon leaving Canada.) 9. While attending social gatherings 10. While using public areas, such as parks and sidewalks 11. While using public transit, such as buses, trains, or taxis 12. Any other situation   (Don't know, Refusal not allowed) |
| **Country of Origin** | Where were you born? | 1. Born in Canada 2. Born outside Canada   (Don't know, Refusal not allowed) |
| **Immigration status** | Are you a Canadian citizen? | 1. Yes, a Canadian citizen by birth 2. Yes, a Canadian citizen by naturalization (Help text: **Canadian citizen by naturalization** refers to an **immigrant** who was granted citizenship of Canada under the *Citizenship Act*.) 3. No, not a Canadian citizen (Don't know, Refusal not allowed) |
| **Immigration status** | Are you a landed immigrant (or permanent resident)? | ON-SCREEN HELP: A landed immigrant or permanent resident is a person who has been granted the right to live in Canada permanently by immigration authorities.   - No - Yes   (Don't know, Refusal not allowed) |
| **Ethnicity/culture** | The following question collects information in accordance with the *Employment Equity Act* and its Regulations and Guidelines to support programs that promote equal opportunity for everyone to share in the social, cultural, and economic life of Canada.  Are you: | ON-SCREEN HELP: Select all that apply.   1. White 2. South Asian (Help text: **e.g.**, East Indian, Pakistani, Sri Lankan) 3. Chinese 4. Black 5. Filipino 6. Arab 7. Latin American 8. Southeast Asian (Help text: **e.g.**, Vietnamese, Cambodian, Laotian, Thai) 9. West Asian (Help text: **e.g.**, Iranian, Afghan) 10. Korean 11. Japanese 12. Other   (Don't know, Refusal not allowed)  Context Sensitive Help:  *Select or specify more than one answer, if applicable, from the list provided.*  *Population group should not be confused with citizenship or nationality.*  *For persons who belong to more than one population group: - select all categories that apply - do not report “bi-racial” or “mixed” in the “Other — specify” box provided* |
| **Indigenous identity** | Are you First Nations, Métis or Inuk (Inuit)? | 1 No  2 Yes, First Nations  3 Yes, Métis  4 Yes, Inuk (Inuit) (Don't know, Refusal not allowed) |

**Supplement 2. Prevalence of situation-based discrimination stratified by race-migration nexus in Canada, ICCED-2020 (N=3,2605)**

|  | CB whites | CB non-whites | FB whites | FB non-whites | Indigenous | Total | X^2^ |
| --- | --- | --- | --- | --- | --- | --- | --- |
|  | n=24412 | n=1865 | n=2300 | n=2887 | n=1141 | n=32605 | P-value |
| **Cumulative discrimination (during pandemic)** | |  |  |  |  |  | <0.001 |
| No exposure (0) | 77.6% | 48.5% | 78.3% | 53.9% | 52.5% | 73.0% |  |
| Have exposure (1-12) | 22.4% | 51.5% | 21.7% | 46.1% | 47.5% | 27.0% |  |
| 1 situation | 11.7% | 18.8% | 12.0% | 18.9% | 14.9% | 12.9% | <0.001 |
| 2 situations | 5.6% | 13.6% | 5.3% | 11.6% | 13.1% | 6.9% |  |
| 3 situations | 2.6% | 8.3% | 2.2% | 7.2% | 7.8% | 3.5% |  |
| ≥ 4 situations | 2.4% | 10.7% | 2.1% | 8.4% | 11.7% | 3.7% |  |
| **Cumulative discrimination (before pandemic)** | |  |  |  |  |  | <0.001 |
| No exposure (0) | 60.8% | 30.6% | 61.9% | 35.3% | 32.6% | 55.9% |  |
| Have exposure (1-12) | 39.2% | 69.4% | 38.1% | 64.7% | 67.4% | 44.1% |  |
| 1 situation | 15.8% | 16.1% | 17.3% | 20.6% | 16.8% | 16.4% | <0.001 |
| 2 situations | 9.2% | 15.5% | 10.9% | 15.9% | 14.7% | 10.5% |  |
| 3 situations | 6.4% | 12.7% | 4.8% | 10.3% | 10.7% | 7.1% |  |
| ≥ 4 situations | 7.8% | 25.0% | 5.2% | 17.8% | 25.2% | 10.1% |  |
| **Discrimination by situation** |  |  |  |  |  |  |  |
| **Store, bank, or restaurant** |  |  |  |  |  |  |  |
| During pandemic | 28.3% | 49.7% | 30.1% | 47.7% | 46.7% | 34.8% | <0.001 |
| Pre pandemic | 29.3% | 51.1% | 28.2% | 45.9% | 48.1% | 34.3% | <0.001 |
| **School (attending school/classes)** |  |  |  |  |  |  |  |
| During pandemic | 4.0% | 7.1% | 3.2% | 6.5% | 7.4% | 4.9% | <0.001 |
| Pre pandemic | 11.1% | 22.9% | 8.6% | 15.8% | 18.7% | 13.0% | <0.001 |
| **On the internet (e.g., social media)** |  |  |  |  |  |  |  |
| During pandemic | 34.9% | 36.4% | 30.3% | 24.2% | 50.0% | 34.1% | <0.001 |
| Pre pandemic | 31.4% | 32.5% | 21.9% | 18.2% | 43.0% | 29.8% | <0.001 |
| **Workplace (Job application/promotion)** | |  |  |  |  |  |  |
| During pandemic | 38.1% | 27.3% | 35.9% | 37.3% | 36.7% | 36.6% | <0.001 |
| Pre pandemic | 54.0% | 50.6% | 49.8% | 57.6% | 52.9% | 53.9% | <0.001 |
| **Housing (buying/renting)** |  |  |  |  |  |  |  |
| During pandemic | 3.7% | 4.5% | 3.6% | 5.5% | 9.8% | 4.4% | <0.001 |
| Pre pandemic | 6.0% | 7.3% | 5.1% | 8.8% | 14.8% | 6.9% | <0.001 |
| **Police (interacting with the police)** |  |  |  |  |  |  |  |
| During pandemic | 3.6% | 8.3% | 3.0% | 8.6% | 13.7% | 5.4% | <0.001 |
| Pre pandemic | 5.0% | 15.8% | 5.4% | 13.4% | 21.5% | 8.0% | <0.001 |
| **Court (interacting with the courts)** |  |  |  |  |  |  |  |
| During pandemic | 1.5% | 2.8% | 2.4% | 2.6% | 4.8% | 2.1% | <0.001 |
| Pre pandemic | 2.2% | 3.6% | 2.7% | 3.7% | 7.2% | 2.8% | <0.001 |
| **Crossing border into Canada** |  |  |  |  |  |  |  |
| During pandemic | 0.9% | 2.4% | 3.0% | 3.5% | 2.8% | 1.7% | <0.001 |
| Pre pandemic | 1.8% | 12.9% | 4.2% | 12.8% | 6.0% | 4.6% | <0.001 |
| **Social gathering** |  |  |  |  |  |  |  |
| During pandemic | 16.0% | 17.8% | 13.3% | 14.5% | 21.8% | 16.2% | <0.001 |
| Pre pandemic | 30.0% | 35.9% | 24.9% | 21.5% | 35.8% | 29.4% | <0.001 |
| **Public areas (e.g., Parks, sidewalks)** | |  |  |  |  |  |  |
| During pandemic | 26.1% | 52.6% | 24.7% | 43.8% | 33.2% | 32.0% | <0.001 |
| Pre pandemic | 29.2% | 46.2% | 22.6% | 35.4% | 37.3% | 31.6% | <0.001 |
| **Public transit (e.g., Buses, trains)** |  |  |  |  |  |  |  |
| During pandemic | 9.6% | 22.5% | 11.4% | 22.8% | 19.2% | 13.7% | <0.001 |
| Pre pandemic | 18.9% | 35.2% | 14.9% | 27.8% | 24.8% | 21.6% | <0.001 |
| **Other situations** |  |  |  |  |  |  |  |
| During pandemic | 26.4% | 16.6% | 24.5% | 16.4% | 24.7% | 23.6% | <0.001 |
| Pre pandemic | 20.5% | 16.9% | 20.0% | 16.3% | 24.1% | 19.8% | <0.001 |

**Notes:** Respondents were able to report more than one perceived reason for discrimination. As such, totals do not equal the sum of the sub-categories. Pre-pandemic= two years before the COVID-19 pandemic; During pandemic=January to August (survey recall period).

**Supplement 3. Prevalence of identity-based discrimination stratified by race-migration nexus in Canada, ICCED-2020 (N=3,2605)**

|  | CB whites | CB non-whites | FB whites | FB non-whites | Indigenous | Total | X^2^ |
| --- | --- | --- | --- | --- | --- | --- | --- |
|  | n=24412 | n=1865 | n=2300 | n=2887 | n=1141 | n=32605 | P-value |
| **Cumulative discrimination (during pandemic)** |  |  |  |  |  |  | <0.001 |
| No exposure (0) | 77.6% | 48.5% | 78.3% | 53.9% | 52.5% | 73.0% |  |
| Have exposure (1-11) | 22.4% | 51.5% | 21.7% | 46.1% | 47.5% | 27.0% |  |
| 1 identity | 13.5% | 17.2% | 12.0% | 15.9% | 16.8% | 13.9% | <0.001 |
| 2 identities | 5.3% | 16.0% | 5.6% | 13.1% | 11.4% | 6.9% |  |
| 3 identities | 2.2% | 9.0% | 2.6% | 8.2% | 9.6% | 3.4% |  |
| ≥ 4 identities | 1.3% | 9.3% | 1.6% | 8.9% | 9.6% | 2.8% |  |
| **Cumulative discrimination (before pandemic)** |  |  |  |  |  |  | <0.001 |
| No exposure (0) | 60.8% | 30.6% | 61.9% | 35.3% | 32.6% | 55.9% |  |
| Have exposure (1-11) | 39.2% | 69.4% | 38.1% | 64.7% | 67.4% | 44.1% |  |
| 1 identity | 20.1% | 15.1% | 18.1% | 17.1% | 19.6% | 19.4% | <0.001 |
| 2 identities | 11.4% | 18.1% | 10.5% | 17.6% | 17.2% | 12.5% |  |
| 3 identities | 5.0% | 14.6% | 5.7% | 12.6% | 13.2% | 6.6% |  |
| ≥ 4 identities | 2.7% | 21.6% | 3.8% | 17.4% | 17.4% | 5.7% |  |
| **Discrimination by identity** |  |  |  |  |  |  |  |
| **Indigenous identity** |  |  |  |  |  |  |  |
| During pandemic | 0.1% | 0.2% | 0.0% | 0.9% | 49.8% | 3.3% | <0.001 |
| Pre pandemic | 0.1% | 0.5% | 0.0% | 0.5% | 52.4% | 3.0% | <0.001 |
| **Ethnicity/Culture** |  |  |  |  |  |  |  |
| During pandemic | 3.6% | 54.4% | 14.1% | 53.4% | 17.5% | 18.1% | <0.001 |
| Pre pandemic | 3.5% | 57.0% | 17.9% | 54.5% | 15.7% | 16.5% | <0.001 |
| **Race/Skin color** |  |  |  |  |  |  |  |
| During pandemic | 5.8% | 74.7% | 6.0% | 73.3% | 20.1% | 24.4% | <0.001 |
| Pre pandemic | 5.0% | 73.8% | 5.7% | 67.6% | 19.2% | 20.1% | <0.001 |
| **Religion** |  |  |  |  |  |  |  |
| During pandemic | 5.0% | 7.3% | 6.0% | 9.6% | 4.8% | 6.0% | <0.001 |
| Pre pandemic | 5.4% | 12.3% | 7.1% | 12.7% | 5.3% | 7.0% | <0.001 |
| **Language** |  |  |  |  |  |  |  |
| During pandemic | 7.5% | 9.4% | 9.4% | 17.1% | 5.0% | 9.1% | <0.001 |
| Pre pandemic | 9.0% | 11.4% | 12.9% | 21.4% | 7.2% | 11.0% | <0.001 |
| **Accent** |  |  |  |  |  |  |  |
| During pandemic | 0.5% | 2.5% | 4.7% | 12.8% | 1.8% | 2.0% | <0.001 |
| Pre pandemic | 1.4% | 4.5% | 12.3% | 25.2% | 3.9% | 4.5% | <0.001 |
| **Physical appearance (weight, clothing)** |  |  |  |  |  |  |  |
| During pandemic | 27.6% | 25.6% | 18.3% | 18.5% | 39.1% | 26.2% | <0.001 |
| Pre pandemic | 35.7% | 36.9% | 20.0% | 21.8% | 47.1% | 33.6% | <0.001 |
| **Sex/sexual orientation/expression** |  |  |  |  |  |  |  |
| During pandemic | 44.6% | 26.1% | 34.5% | 13.6% | 36.3% | 36.8% | <0.001 |
| Pre pandemic | 59.4% | 45.8% | 46.4% | 26.0% | 50.3% | 52.6% | <0.001 |
| **Age** |  |  |  |  |  |  |  |
| During pandemic | 29.0% | 17.8% | 33.9% | 13.1% | 28.4% | 25.6% | <0.001 |
| Pre pandemic | 31.4% | 29.5% | 33.9% | 16.5% | 32.9% | 29.5% | <0.001 |
| **Disability (mental/physical)** |  |  |  |  |  |  |  |
| During pandemic | 23.5% | 8.9% | 15.5% | 4.2% | 24.9% | 18.6% | <0.001 |
| Pre pandemic | 18.2% | 9.4% | 11.2% | 4.7% | 21.8% | 15.4% | <0.001 |
| **Other reasons** |  |  |  |  |  |  |  |
| During pandemic | 16.7% | 6.7% | 16.9% | 8.9% | 16.8% | 14.5% | <0.001 |
| Pre pandemic | 7.7% | 4.6% | 8.0% | 5.4% | 8.2% | 7.1% | <0.001 |

**Notes:** Respondents were able to report more than one perceived reason for discrimination. As such, totals do not equal the sum of the sub-categories. Pre-pandemic= two years before the COVID-19 pandemic; During pandemic=January to August (survey recall period).

**Supplement 4. Multinomial Logistic regression estimating likelihood of perceived discrimination before and during the pandemic by race-migration nexus in Canada, ICCED-2020 (N=3,2605)**

|  | **CB non-White** | | | |  | **FB White** | | | |  | **FB non-White** | | | |  | **Indigenous** | | | |
| --- | --- | --- | --- | --- | --- | --- | --- | --- | --- | --- | --- | --- | --- | --- | --- | --- | --- | --- | --- |
|  | **(vs. CB White)** | | | |  | **(vs. CB White)** | | | |  | **(vs. CB White)** | | | |  | **(vs. CB White)** | | | |
| **Outcome variables** | **OR** | **99% CI** | | **Sig.** |  | **OR** | **99% CI** | | **Sig.** |  | **OR** | **99% CI** | | **Sig.** |  | **OR** | **99% CI** | | **Sig.** |
| **Unadjusted (crude) models** | | | | | | | | | | | | | | | | | | | |
| **Discrimination (yes/no)** |  |  |  |  |  |  |  |  |  |  |  |  |  |  |  |  |  |  |  |
| Discrimination during pandemic | **3.65** | **3.32** | **4.02** | **<0.001** | | 0.95 | 0.86 | 1.06 | 0.344 |  | **3.00** | **2.76** | **3.25** | **<0.001** | | **3.13** | **2.77** | **3.53** | **<0.001** |
| Discrimination pre-pandemic | **3.51** | **3.17** | **3.89** | **<0.001** | | 0.95 | 0.87 | 1.04 | 0.258 |  | **2.90** | **2.67** | **3.15** | **<0.001** | | **3.16** | **2.78** | **3.59** | **<0.001** |
| **Discrimination addictive scale** |  |  |  |  |  |  |  |  |  |  |  |  |  |  |  |  |  |  |  |
| **Discrimination during pandemic (Ref. 0)** | |  |  |  |  |  |  |  |  |  |  |  |  |  |  |  |  |  |  |
| 1 situation | **2.61** | **2.20** | **3.10** | **<0.001** | | 1.03 | 0.86 | 1.24 | 0.655 |  | **2.40** | **2.08** | **2.76** | **<0.001** | | **2.09** | **1.67** | **2.62** | **<0.001** |
| 2 situations | **4.27** | **3.50** | **5.21** | **<0.001** | | 0.91 | 0.70 | 1.20 | 0.394 |  | **3.30** | **2.77** | **3.92** | **<0.001** | | **3.51** | **2.72** | **4.53** | **<0.001** |
| 3 situations | **5.83** | **4.54** | **7.48** | **<0.001** | | 0.83 | 0.55 | 1.25 | 0.243 |  | **4.29** | **3.42** | **5.37** | **<0.001** | | **3.96** | **2.81** | **5.58** | **<0.001** |
| ≥ 4 situations | **7.49** | **5.89** | **9.54** | **<0.001** | | 0.86 | 0.56 | 1.32 | 0.376 |  | **5.31** | **4.26** | **6.62** | **<0.001** | | **7.98** | **6.04** | **10.53** | **<0.001** |
| **Discrimination pre-pandemic (Ref. 0)** | |  |  |  |  |  |  |  |  |  |  |  |  |  |  |  |  |  |  |
| 1 situation | **1.99** | **1.65** | **2.41** | **<0.001** | | **1.13** | 0.97 | 1.32 | 0.037 |  | **2.17** | **1.88** | **2.49** | **<0.001** | | **1.97** | **1.57** | **2.48** | **<0.001** |
| 2 situations | **3.45** | **2.85** | **4.19** | **<0.001** | | 1.11 | 0.91 | 1.35 | 0.175 |  | **3.00** | **2.56** | **3.50** | **<0.001** | | **2.86** | **2.23** | **3.66** | **<0.001** |
| 3 situations | **4.09** | **3.31** | **5.05** | **<0.001** | | **0.70** | **0.53** | **0.93** | **0.001** |  | **2.72** | **2.25** | **3.28** | **<0.001** | | **3.08** | **2.33** | **4.07** | **<0.001** |
| ≥ 4 situations | **6.47** | **5.44** | **7.70** | **<0.001** | | **0.62** | **0.48** | **0.82** | **<0.001** | | **3.95** | **3.38** | **4.60** | **<0.001** | | **5.83** | **4.71** | **7.21** | **<0.001** |
| **Discrimination during pandemic (Ref. 0)** | |  |  |  |  |  |  |  |  |  |  |  |  |  |  |  |  |  |  |
| 1 identity | **2.04** | **1.71** | **2.42** | **<0.001** | | 0.88 | 0.74 | 1.04 | 0.049 |  | **1.69** | **1.46** | **1.96** | **<0.001** | | **1.84** | **1.48** | **2.29** | **<0.001** |
| 2 identities | **4.79** | **3.98** | **5.78** | **<0.001** | | 1.03 | 0.81 | 1.32 | 0.752 |  | **3.54** | **3.00** | **4.18** | **<0.001** | | **3.14** | **2.43** | **4.08** | **<0.001** |
| 3 identities | **6.53** | **5.12** | **8.34** | **<0.001** | | 1.15 | 0.81 | 1.65 | 0.310 |  | **5.38** | **4.35** | **6.65** | **<0.001** | | **6.45** | **4.83** | **8.62** | **<0.001** |
| ≥ 4 identities | **11.02** | **8.52** | **14.25** | **<0.001** | | 1.15 | 0.73 | 1.82 | 0.426 |  | **9.55** | **7.63** | **11.96** | **<0.001** | | **10.57** | **7.81** | **14.31** | **<0.001** |
| **Discrimination pre-pandemic (Ref. 0)** | | |  |  |  |  |  |  |  |  |  |  |  |  |  |  |  |  |  |
| 1 identity | **1.48** | **1.22** | **1.80** | **<0.001** | | 0.88 | 0.76 | 1.03 | 0.033 |  | **1.46** | **1.26** | **1.69** | **<0.001** | | **1.81** | **1.45** | **2.26** | **<0.001** |
| 2 identities | **3.15** | **2.62** | **3.79** | **<0.001** | | 0.90 | 0.75 | 1.09 | 0.156 |  | **2.65** | **2.28** | **3.07** | **<0.001** | | **2.80** | **2.22** | **3.54** | **<0.001** |
| 3 identities | **5.83** | **4.75** | **7.15** | **<0.001** | | 1.12 | 0.88 | 1.44 | 0.231 |  | **4.36** | **3.66** | **5.19** | **<0.001** | | **4.93** | **3.80** | **6.40** | **<0.001** |
| ≥ 4 identities | **15.96** | **13.11** | **19.43** | **<0.001** | | **1.40** | **1.04** | **1.89** | **0.004** |  | **11.16** | **9.38** | **13.28** | **<0.001** | | **12.00** | **9.36** | **15.39** | **<0.001** |
| **Fully adjusted models** | | | | | | | | | | | | | | | | | | | |
| **Discrimination (yes/no)** |  |  |  |  |  |  |  |  |  |  |  |  |  |  |  |  |  |  |  |
| Discrimination during pandemic | **3.93** | **3.53** | **4.37** | **<0.001** | | 1.06 | 0.95 | 1.19 | 0.298 |  | **4.01** | **3.64** | **4.42** | **<0.001** | | **2.40** | **2.10** | **2.75** | **<0.001** |
| Discrimination pre-pandemic | **3.39** | **3.03** | **3.79** | **<0.001** | | 1.07 | 0.97 | 1.18 | 0.205 |  | **3.74** | **3.39** | **4.13** | **<0.001** | | **2.63** | **2.29** | **3.03** | **<0.001** |
| **Discrimination addictive scale** |  |  |  |  |  |  |  |  |  |  |  |  |  |  |  |  |  |  |  |
| **Discrimination during pandemic (Ref. 0)** | |  |  |  |  |  |  |  |  |  |  |  |  |  |  |  |  |  |  |
| 1 situation | **2.61** | **2.18** | **3.13** | **<0.001** | | 1.06 | 0.88 | 1.27 | 0.438 |  | **2.69** | **2.30** | **3.16** | **<0.001** | | **1.86** | **1.48** | **2.34** | **<0.001** |
| 2 situations | **4.35** | **3.52** | **5.37** | **<0.001** | | 0.97 | 0.74 | 1.28 | 0.799 |  | **4.21** | **3.45** | **5.13** | **<0.001** | | **2.88** | **2.21** | **3.75** | **<0.001** |
| 3 situations | **5.92** | **4.54** | **7.71** | **<0.001** | | 0.91 | 0.60 | 1.38 | 0.541 |  | **5.53** | **4.27** | **7.17** | **<0.001** | | **3.10** | **2.18** | **4.42** | **<0.001** |
| ≥ 4 situations | **7.50** | **5.77** | **9.73** | **<0.001** | | 1.00 | 0.64 | 1.55 | 0.994 |  | **7.20** | **5.57** | **9.31** | **<0.001** | | **5.96** | **4.44** | **8.00** | **<0.001** |
| **Discrimination during pandemic (Ref. 0)** | | |  |  |  |  |  |  |  |  |  |  |  |  |  |  |  |  |  |
| 1 identity | **2.08** | **1.73** | **2.49** | **<0.001** | | 0.92 | 0.77 | 1.10 | 0.203 |  | **1.99** | **1.69** | **2.34** | **<0.001** | | **1.62** | **1.29** | **2.03** | **<0.001** |
| 2 identities | **4.81** | **3.94** | **5.87** | **<0.001** | | 1.09 | 0.85 | 1.41 | 0.363 |  | **4.33** | **3.58** | **5.24** | **<0.001** | | **2.61** | **2.00** | **3.42** | **<0.001** |
| 3 identities | **6.87** | **5.29** | **8.91** | **<0.001** | | 1.27 | 0.88 | 1.83 | 0.095 |  | **7.43** | **5.82** | **9.47** | **<0.001** | | **5.06** | **3.74** | **6.84** | **<0.001** |
| ≥ 4 identities | **11.78** | **8.92** | **15.54** | **<0.001** | | 1.24 | 0.78 | 1.98 | 0.230 |  | **12.72** | **9.80** | **16.51** | **<0.001** | | **8.17** | **5.94** | **11.23** | **<0.001** |
| **Discrimination pre-pandemic (Ref. 0)** | |  |  |  |  |  |  |  |  |  |  |  |  |  |  |  |  |  |  |
| 1 situation | **1.97** | **1.62** | **2.39** | **<0.001** | | 1.14 | 0.97 | 1.33 | 0.035 |  | **2.34** | **2.00** | **2.75** | **<0.001** | | **1.87** | **1.48** | **2.36** | **<0.001** |
| 2 situations | **3.21** | **2.62** | **3.93** | **<0.001** | | 1.15 | 0.94 | 1.40 | 0.080 |  | **3.43** | **2.87** | **4.11** | **<0.001** | | **2.65** | **2.05** | **3.42** | **<0.001** |
| 3 situations | **3.84** | **3.07** | **4.80** | **<0.001** | | 0.77 | 0.58 | 1.03 | 0.021 |  | **3.52** | **2.84** | **4.37** | **<0.001** | | **2.74** | **2.05** | **3.67** | **<0.001** |
| ≥ 4 situations | **6.24** | **5.13** | **7.58** | **<0.001** | | **0.74** | **0.55** | **0.98** | **0.006** |  | **5.94** | **4.93** | **7.17** | **<0.001** | | **4.84** | **3.81** | **6.15** | **<0.001** |
| **Discrimination pre-pandemic (Ref. 0)** | |  |  |  |  |  |  |  |  |  |  |  |  |  |  |  |  |  |  |
| 1 identity | **1.46** | **1.20** | **1.78** | **<0.001** | | 0.93 | 0.80 | 1.09 | 0.242 |  | **1.70** | **1.44** | **2.01** | **<0.001** | | **1.71** | **1.36** | **2.15** | **<0.001** |
| 2 identities | **3.09** | **2.54** | **3.75** | **<0.001** | | 0.96 | 0.79 | 1.17 | 0.581 |  | **3.32** | **2.79** | **3.95** | **<0.001** | | **2.60** | **2.04** | **3.32** | **<0.001** |
| 3 identities | **5.98** | **4.80** | **7.45** | **<0.001** | | 1.27 | 0.98 | 1.65 | 0.016 |  | **6.24** | **5.09** | **7.65** | **<0.001** | | **4.39** | **3.34** | **5.78** | **<0.001** |
| ≥ 4 identities | **16.88** | **13.57** | **21.00** | **<0.001** | | **1.65** | **1.20** | **2.25** | **<0.001** | | **16.89** | **13.71** | **20.80** | **<0.001** | | **10.16** | **7.76** | **13.30** | **<0.001** |
| **Repeated discrimination (Ref.0)** |  |  |  |  | |  |  |  |  | |  |  |  |  | |  |  |  |  |
| Exposure before COVID-19 only | **2.22** | **1.84** | **2.67** | **<0.001** | | 1.01 | 0.86 | 1.18 | 0.91 | | **2.58** | **2.20** | **3.02** | **<0.001** | | **1.92** | **1.53** | **2.41** | **<0.001** |
| Exposure during COVID-19 only | **3.50** | **2.45** | **4.99** | **<0.001** | | 0.94 | 0.64 | 1.40 | 0.70 | | **4.08** | **3.02** | **5.51** | **<0.001** | | 1.09 | 0.57 | 2.09 | 0.74 |
| Repeated exposure | **5.06** | **4.29** | **5.97** | **<0.001** | | 1.01 | 0.86 | 1.18 | 0.92 | | **4.94** | **4.27** | **5.73** | **<0.001** | | **3.22** | **2.63** | **3.93** | **<0.001** |

Notes: Fully adjusted models were controlled for age, sex, education, household living arrangement, sexual orientation, marital status, language spoken at home, barriers to health care, community belonging. To account for multiple testing, a more stringent criterion was adopted: a significance level of 0.01 (p < 0.01) was considered statistically significant and 99% confidence intervals (99% CI) were used. Given the non-probabilistic nature of the crowdsourcing data in this survey, confidence intervals should be interpreted with extreme caution. Pre-pandemic= two years before the COVID-19 pandemic; During pandemic=January to August 2020 (survey recall period).

**Supplement 5. Logistic regression estimating likelihood of long-term health conditions by discrimination in Canada, ICCED-2020 (N=3,2605)**

|  | Model A | | |  | Model C | | |  | Model C | | |  | Model D | | |  | Model D | | |  | Model E | | |
| --- | --- | --- | --- | --- | --- | --- | --- | --- | --- | --- | --- | --- | --- | --- | --- | --- | --- | --- | --- | --- | --- | --- | --- |
|  | Seeing  (vs. no) | | |  | Hearing  (vs. no) | | |  | Physical  (vs. no) | | |  | Cognitive  (vs. no) | | |  | Mental  (vs. no) | | |  | Other conditions  (vs. no) | | |
| **Explanatory variables** | OR | 99% CI | |  | OR | 99% CI | |  | OR | 99% CI | |  | OR | 99% CI | |  | OR | 99% CI | |  | OR | 99% CI | |
| **During the pandemic** | | | | | | | |  |  |  |  |  |  |  |  |  |  |  |  |  |  |  |  |
| **Regression 1: Discrimination during pandemic (Ref. 0)** | | | | | | | |  |  |  |  |  |  |  |  |  |  |  |  |  |  |  |  |
| 1 identity | 1.63 | 1.41 | 1.88 |  | 1.83 | 1.41 | 2.37 |  | 1.66 | 1.45 | 1.90 |  | 1.81 | 1.58 | 2.08 |  | 1.82 | 1.66 | 2.00 |  | 1.60 | 1.49 | 1.73 |
| 2 identities | 1.97 | 1.64 | 2.36 |  | 2.47 | 1.78 | 3.42 |  | 2.54 | 2.14 | 3.01 |  | 2.66 | 2.25 | 3.14 |  | 2.17 | 1.90 | 2.47 |  | 1.82 | 1.64 | 2.02 |
| 3 identities | 2.33 | 1.84 | 2.94 |  | 2.59 | 1.66 | 4.05 |  | 2.77 | 2.21 | 3.47 |  | 3.04 | 2.45 | 3.76 |  | 2.55 | 2.13 | 3.05 |  | 1.98 | 1.72 | 2.28 |
| ≥ 4 identities | 2.99 | 2.34 | 3.82 |  | 4.45 | 2.90 | 6.82 |  | 3.87 | 3.03 | 4.95 |  | 3.79 | 3.02 | 4.77 |  | 3.41 | 2.79 | 4.17 |  | 3.06 | 2.62 | 3.57 |
| **Regression 2: Discrimination during pandemic (Ref. 0)** | | | | | | | |  |  |  |  |  |  |  |  |  |  |  |  |  |  |  |  |
| 1 situation | 1.62 | 1.40 | 1.89 |  | 1.70 | 1.29 | 2.24 |  | 1.68 | 1.46 | 1.94 |  | 2.00 | 1.74 | 2.30 |  | 1.89 | 1.71 | 2.09 |  | 1.60 | 1.44 | 1.77 |
| 2 situations | 1.77 | 1.47 | 2.14 |  | 2.47 | 1.79 | 3.42 |  | 2.20 | 1.85 | 2.62 |  | 2.16 | 1.82 | 2.56 |  | 2.06 | 1.81 | 2.34 |  | 1.80 | 1.57 | 2.05 |
| 3 situations | 2.11 | 1.67 | 2.68 |  | 2.99 | 1.98 | 4.51 |  | 2.70 | 2.16 | 3.38 |  | 2.69 | 2.17 | 3.33 |  | 2.45 | 2.05 | 2.91 |  | 2.11 | 1.76 | 2.52 |
| ≥ 4 situations | 3.24 | 2.62 | 4.00 |  | 3.71 | 2.51 | 5.48 |  | 3.63 | 2.94 | 4.48 |  | 3.44 | 2.82 | 4.20 |  | 2.71 | 2.27 | 3.22 |  | 2.46 | 2.06 | 2.92 |
| **Two years before the pandemic** | | | | | | |  |  |  |  |  |  |  |  |  |  |  |  |  |  |  |  |  |
| **Regression 3: Discrimination pre-pandemic (Ref. 0)** | | | | | | |  |  |  |  |  |  |  |  |  |  |  |  |  |  |  |  |  |
| 1 identity | 1.60 | 1.38 | 1.84 |  | 1.48 | 1.15 | 1.91 |  | 1.59 | 1.39 | 1.82 |  | 1.79 | 1.55 | 2.06 |  | 1.75 | 1.60 | 1.92 |  | 1.46 | 1.33 | 1.61 |
| 2 identities | 1.86 | 1.58 | 2.19 |  | 1.85 | 1.38 | 2.49 |  | 2.29 | 1.97 | 2.67 |  | 2.24 | 1.91 | 2.62 |  | 2.27 | 2.04 | 2.52 |  | 1.84 | 1.65 | 2.06 |
| 3 identities | 2.32 | 1.90 | 2.82 |  | 1.92 | 1.30 | 2.85 |  | 2.84 | 2.35 | 3.43 |  | 3.27 | 2.73 | 3.92 |  | 2.89 | 2.52 | 3.31 |  | 2.27 | 1.97 | 2.62 |
| ≥ 4 identities | 2.94 | 2.39 | 3.62 |  | 4.11 | 2.86 | 5.90 |  | 3.39 | 2.75 | 4.17 |  | 4.65 | 3.84 | 5.63 |  | 3.67 | 3.15 | 4.28 |  | 2.85 | 2.44 | 3.34 |
| **Regression 4: Discrimination pre-pandemic (Ref. 0)** | | | | | | | |  |  |  |  |  |  |  |  |  |  |  |  |  |  |  |  |
| 1 situation | 1.56 | 1.34 | 1.82 |  | 1.39 | 1.06 | 1.83 |  | 1.54 | 1.33 | 1.78 |  | 1.81 | 1.56 | 2.11 |  | 1.72 | 1.56 | 1.89 |  | 1.46 | 1.32 | 1.62 |
| 2 situations | 1.71 | 1.43 | 2.04 |  | 1.84 | 1.34 | 2.51 |  | 2.11 | 1.80 | 2.49 |  | 2.27 | 1.92 | 2.68 |  | 2.11 | 1.89 | 2.36 |  | 1.72 | 1.53 | 1.94 |
| 3 situations | 2.15 | 1.77 | 2.62 |  | 1.99 | 1.38 | 2.89 |  | 2.49 | 2.07 | 3.00 |  | 2.50 | 2.08 | 3.00 |  | 2.53 | 2.23 | 2.88 |  | 1.99 | 1.73 | 2.28 |
| ≥ 4 situations | 2.73 | 2.29 | 3.24 |  | 3.07 | 2.24 | 4.20 |  | 3.24 | 2.74 | 3.83 |  | 3.43 | 2.92 | 4.02 |  | 3.14 | 2.79 | 3.54 |  | 2.49 | 2.20 | 2.82 |
| **Regression 5: Repeated exposure to discrimination (Ref. 0)** | | | | | | | | | |  |  |  |  |  |  |  |  |  |  |  |  |  |  |
| Exposure before COVID-19 only | 1.53 | 1.32 | 1.78 |  | 1.33 | 1.00 | 1.76 |  | 1.56 | 1.35 | 1.80 |  | 1.82 | 1.57 | 2.10 |  | 1.80 | 1.65 | 1.98 |  | 1.50 | 1.36 | 1.66 |
| Exposure during COVID-19 only | 1.61 | 1.16 | 2.23 |  | 2.26 | 1.35 | 3.78 |  | 1.52 | 1.10 | 2.09 |  | 2.11 | 1.55 | 2.88 |  | 1.73 | 1.40 | 2.15 |  | 1.66 | 1.33 | 2.07 |
| Repeated exposure | 2.28 | 2.00 | 2.61 |  | 2.44 | 1.93 | 3.08 |  | 2.60 | 2.29 | 2.95 |  | 3.01 | 2.64 | 3.43 |  | 2.70 | 2.47 | 2.95 |  | 2.13 | 1.94 | 2.34 |

Note: All models adjusted for race-migration nexus, age, sex, education, household living arrangement, sexual orientation, marital status, language spoken at home, barriers to health care, community belonging. To account for multiple testing, a more stringent criterion was adopted: a significance level of 0.01 (p < 0.01) was considered statistically significant and 99% confidence intervals (99% CI) were used. Pre-pandemic= two years before the COVID-19 pandemic; During pandemic=January to August 2020 (survey recall period). Given the non-probabilistic nature of the crowdsourcing data in this survey, measures of precision (e.g., confidence intervals) should be interpreted with extreme caution.
